# Structural Ensemble of the Insulin Monomer

**DOI:** 10.1021/acs.biochem.1c00583

**Published:** 2021-10-12

**Authors:** Luis Busto-Moner, Chi-Jui Feng, Adam Antoszewski, Andrei Tokmakoff, Aaron R. Dinner

**Affiliations:** † Department of Chemistry, The University of Chicago, Chicago, Illinois 60637, United States; ‡ James Franck Institute, The University of Chicago, Chicago, Illinois 60637, United States; § Institute for Biophysical Dynamics, The University of Chicago, Chicago, Illinois 60637, United States

## Abstract

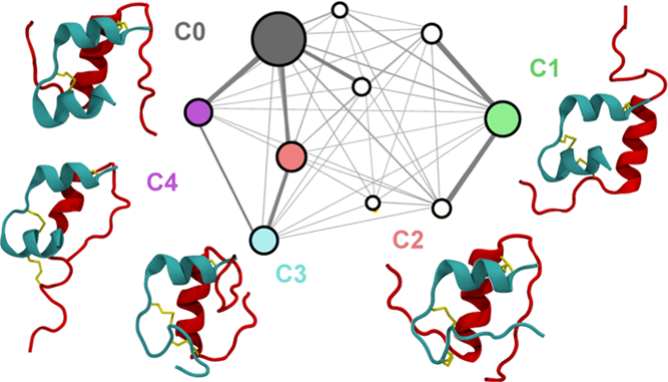

Experimental evidence suggests that monomeric insulin exhibits significant conformational heterogeneity, and modifications of apparently disordered regions affect both biological activity and the longevity of pharmaceutical formulations, presumably through receptor binding and fibrillation/degradation, respectively. However, a microscopic understanding of conformational heterogeneity has been lacking. Here, we integrate all-atom molecular dynamics simulations with an analysis pipeline to investigate the structural ensemble of human insulin monomers. We find that 60% of the structures present at least one of the following elements of disorder: melting of the A-chain N-terminal helix, detachment of the B-chain N-terminus, and detachment of the B-chain C-terminus. We also observe partial melting and extension of the B-chain helix and significant conformational heterogeneity in the region containing the B-chain β-turn. We then estimate hydrogen-exchange protection factors for the sampled ensemble and find them in line with experimental results for KP-insulin, although the simulations underestimate the importance of unfolded states. Our results help explain the ready exchange of specific amide sites that appear to be protected in crystal structures. Finally, we discuss the implications for insulin function and stability.

## Introduction

The protein hormone insulin regulates glucose uptake in cells, and a lack of its activity due to insufficiency or insensitivity leads to diabetes mellitus, a widespread life-threatening metabolic disease; administration of insulin and its analogues is a key treatment.^1,2^ Insulin is predominantly hexameric and dimeric during production, delivery, and circulation, but it binds to its receptor as a monomer,^3^ in a conformation that differs significantly from that in solution NMR and X-ray crystallographic structures.^4,5^ Conditions and sequence changes that favor the monomeric form promote fibrillation^6−9^ and degradation,^10^ which limit the stability of therapeutic preparations. An understanding of the structures accessible to the monomer under physiological conditions is therefore of great interest in medicine, biophysics, and structural biology.

**Figure 1:**
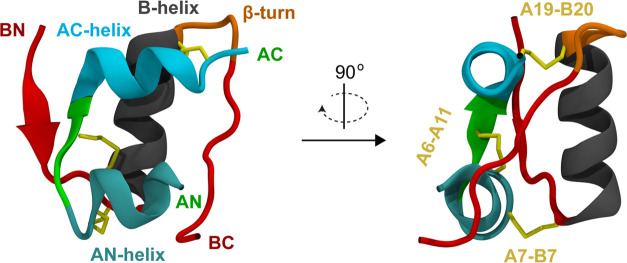
Schematic view of the insulin structure (PDB ID 2JV1^11^). A-chain N- and C-termini are labeled in green (AN and AC), and B-chain N- and C-termini are labeled in red (BN and BC). The A-chain α-helices (AN, Gly^A1^-Ser^A9^; AC, Leu^A13^-Cys^A20^) are colored teal and cyan, respectively. The B-helix (Gly^B9^-Cys^B19^) is shown in black. The Gly^B20^-Gly^B23^ β-turn is shown in orange. Disulfide bridges are shown in yellow.

Available structures for the insulin monomer (e.g., Figure 1, from NMR in water/acetonitrile^11^) are consistent with the T-state structure observed in crystallographic studies of the hexamer.^12^ In the monomer, there are 51 residues distributed between two chains, denoted A (21 residues) and B (30 residues). Two disulfide bridges (Cys^A7^-Cys^B7^ and Cys^A20^-Cys^B19^) connect the two chains, and a third (Cys^A6^-Cys^A11^) is internal to the A chain. The secondary structure of the T state consists of two α-helices in the A-chain, one at the N-terminus (Gly^A1^-Ser^A9^) and one at the C-terminus (Leu^A13^-Cys^A20^); we term these the AN- and AC-helices, respectively. The B-chain includes one α-helix (Gly^B9^-Cys^B19^), which we term the B-helix, and a β-turn (Gly^B20^-Gly^B23^).

Measurements of amide hydrogen exchange for KP-insulin,^13,14^ an analogue that is monomeric in solution, show little protection at the A-chain N-terminus (A1–A9), the B-chain N-terminus (Phe^B1^-Cys^B7^), and the B-chain C-terminus (Gly^B20^-Ala^B30^).^15^ The exchange at these sites indicates that these segments sample solvent-exposed states, though the nature of these states remains unclear. Experimental characterization of the wild-type monomer structural ensemble itself is hindered by both fibrillation and oligomerization at concentrations ranging from micromolar to millimolar.^16^ Experimental structural information about the monomer thus mainly comes from studies of sequences with substitutions, as above, or from studies in solution conditions that differ significantly from physiological ones (e.g., pH < 3 and/or with cosolvents),^7,11,13,14^ which can impact the degree of disorder.^17^

Setting aside the issues above, structural characterization of a protein with disordered regions is experimentally challenging because conformations can interconvert on pico- to millisecond lifetimes.^18−20^ Although NMR relaxation measurements can access these timescales to give information about contributing motions, extracting the structures and their populations from chemical shifts is not straightforward due to averaging.^21^ Measurements of Förster resonance energy transfer between dye labels can access such timescales for single molecules,^22−24^ but they are impractical for small proteins with sizes smaller than typical transfer radii, on the scale of the labels themselves.

Simulations can provide direct access to microscopic structures and the forces stabilizing them, but there have been surprisingly few all-atom molecular dynamics studies of the insulin monomer. Nanosecond-scale unbiased simulations of the porcine insulin monomer starting from the T state in the hexamer crystal structure^12^ showed disorder in the N- and C-termini of the B-chain^25^ but not the AN-helix. The root-mean-square deviation from the T state suggests significant unfolding also in ref (26), which takes a similar approach, though few structural details are given. Bias-exchange metadynamics simulations of porcine insulin at low pH and high temperature produced a diverse ensemble of essentially fully unfolded states.^27^

**Figure 2:**
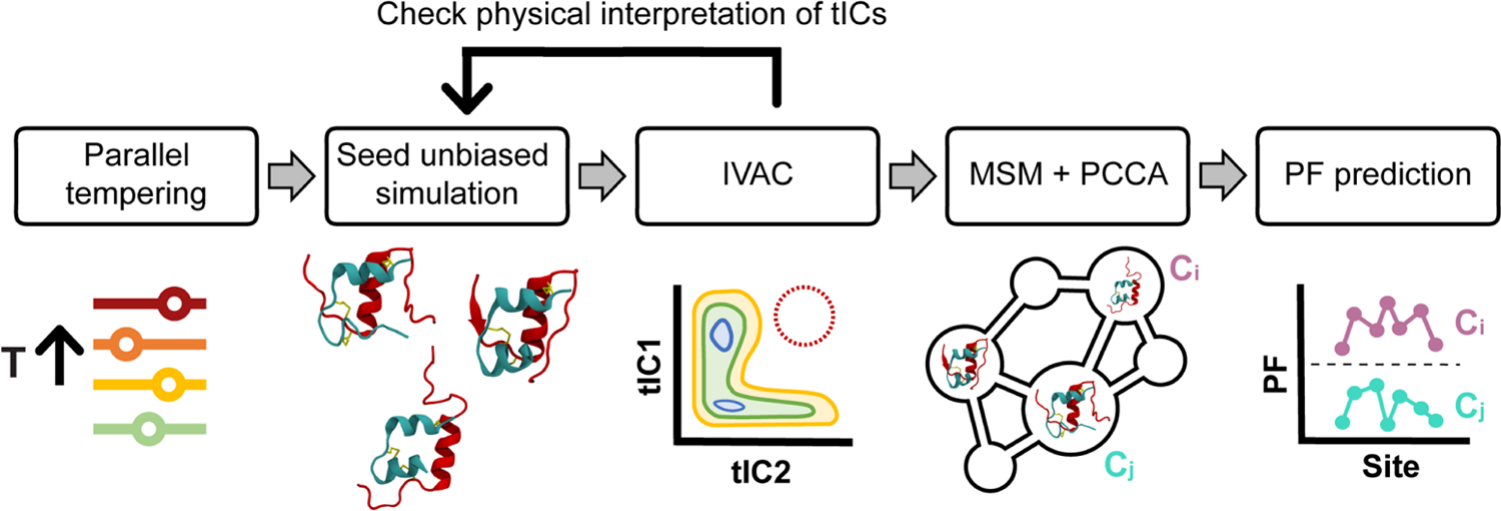
Overview of the pipeline used to sample and cluster structures. The resulting clusters were used to model the protection factors of individual H_N_ sites.

Here, we report a study of the structural ensemble of human wild-type insulin at low pH and room temperature. We combine enhanced sampling,^28,29^ extensive unbiased simulation, and a variety of analysis methods^30−34^ to ensure a good exploration of the accessible conformational space (Figure 2). The simulations indicate that 60% of the population under these conditions contains at least one of the following elements: melting of the AN-helix, detachment of the B-chain N-terminus, and detachment of the B-chain C-terminus. We further characterize the B-chain conformations with regard to the structure of the B-helix and the β-turn, as well as the timescales for interconversion between states, which are on the order of microseconds. Our results are consistent with the hydrogen–deuterium exchange results for KP-insulin described above,^15^ and we use simulated protection factors (PFs) to further assess the contributions from different states. Finally, we discuss how our atomic-resolution description of conformation heterogeneity within the insulin monomer structural ensemble provides insights into insulin-receptor binding and fibrillation.

## Materials and Methods

The main goal of this work was to explore the conformational space of the insulin monomer. The pipeline we followed is summarized in Figure 2. A first set of structures was generated using parallel tempering,^28,29^ which was used to seed equilibrium unbiased simulations. The slowest relaxing motions of the protein were then identified using the integrated variational approach for conformational dynamics (IVAC),^32,33^ and low probability structures in that space were subsequently sampled as starting points of new unbiased simulations. The resulting data set was then clustered into 1000 states to build a Markov state model (MSM),^30,31^ which was further coarse-grained using Perron cluster analysis (PCCA).^34^ The resulting 10 clusters characterize the insulin monomer structural ensemble. We used these clusters to estimate the individual H_N_ protection factors for comparison with experimental hydrogen-exchange data.^15^

### System Setup and Equilibration

The system was modeled with the CHARMM36m force field.^35−37^ All simulations were performed using GROMACS 5.1.4,^38^ and the system was prepared using CHARMM-GUI 2.1;^39,40^ All molecular visualizations were done in VMD.^41^ Unless otherwise noted, simulations were carried out in the isochoric isothermal (NVT) ensemble at 303.15 K using a Langevin thermostat^42^ with a 2-fs time step and a friction constant of 10 ps^–1^ applied to all atoms. All bonds to hydrogen atoms were constrained using the LINCS algorithm.^43^ Periodic boundary conditions were employed, and the particle-mesh Ewald method^44^ was used to calculate electrostatic forces with a cutoff distance of 1.2 nm. The Lennard-Jones interactions were smoothly switched off from 1.0 to 1.2 nm through the built-in GROMACS force-switch function.

The starting point for our simulations was an NMR structure of human insulin in water/acetonitrile solution (PDB ID 2JV1).^11^ Hydrogens were added to the PDB structure, and it was solvated in a (7 nm)^3^ box using TIP3P water.^45^ PROPKA^46,47^ was used to estimate the protonation state of individual residues at pH 2.5, within the pH range commonly used to better solubilize insulin for NMR and IR experiments. Given the resulting charge states, 4 Cl^–^ ions were added to neutralize the system. There were 25 006 atoms in total.

The system was energetically minimized using the steepest descent method until the maximum force felt by the system was below 1000 kJ/mol nm. The system was then equilibrated for 100 ps in the NVT ensemble with a 1-fs time step, followed by 10 ns in the NPT ensemble at 1 bar using the Parrinello–Rahman barostat,^48^ with a 2-fs time step and time constant of 5.0 ps. For the energy minimization and equilibration above, harmonic restraints were used to stabilize the positions of all nonhydrogen protein atoms. The system was equilibrated further for 1 ns in the NPT ensemble without position restraints. We then imposed a box size of (6.5 nm)^3^, based on the average size of the highest temperature simulations in the parallel tempering (see below). This box size was used for all further simulations, including the unbiased ones. The system was equilibrated once more without position restraints for 1 ns in the NVT ensemble.

### Parallel Tempering

In the absence of a priori knowledge of structural descriptors that could resolve different stable states, we chose to use parallel tempering to enhance conformational exploration.^28,29^ The goal of these simulations was to generate a diverse set of conformations to further seed independent room temperature simulations. A first set of 20 replicas ranging from 303 to 360 K was used, with temperatures spaced to achieve an exchange probability of 5% based on the number of atoms.^49^ Before production runs, each simulation was individually equilibrated at the desired temperature following the procedure detailed above. As exchanging replicas must have the same box size, before the final NVT equilibration, we set the box size to the average for the highest temperature simulations (6.5 nm)^3^. The total length of each individual simulation was 100 ns, with exchanges attempted every 1 ps. Briefly, 19% of exchanges were accepted. A second set of 20 replicas between 350 and 415 K was run, with individual simulations lasting 200 ns and the same exchange attempt rate. Typically, 23% of exchanges were accepted. From these data, we extracted 15 structures by hand that were representative of the elements of disorder observed and used them to seed free simulations at 303.15 K for 2.8 μs.

### IVAC

To characterize and visualize the structures, we projected them onto the slowest decorrelating modes of the system identified by a modified version of the variational approach to conformational analysis (VAC).^32^ The resulting time-lagged independent components (tICs) are linear combinations of the input features. Common feature choices are atom positions, distances between selected atoms, and (pseudo)dihedral angles. Here, we used pairwise distances between the α-carbons of Gly^A1^, Cys^A6^, Tyr^A19^, Val^B2^, Ser^B9^, Tyr^B16^, Gly^B20^, Phe^B24^, and Ala^B30^. The idea was to cover the whole protein and all of the observed conformational changes with a reduced initial number of dimensions (36).

In VAC, a lag time needs to be chosen to compute the time-correlation matrix between input dimensions. The lag time should be long enough for fast processes to decorrelate but short enough to resolve the dynamics of interest. In practice, choosing this lag time can be difficult, and the results can be sensitive to it. We overcome this issue in the present study using integrated VAC (IVAC), which sums the time-correlation matrices over a range of lag times for greater robustness.^33^

We iteratively applied IVAC with lag times ranging from 1 to 100 ns to develop a data set with good coverage of the accessible space. First, we applied it to the 15 unbiased 2.8-μs trajectories from the parallel-tempering seeds; we projected our sampling onto the resulting first three tICs and selected 14 structures from poorly sampled regions to seed additional 2.8-μs simulations. We repeated this process (IVAC on the cumulative data set to determine tICS, projection, selection of seed structures, and unbiased simulation), selecting 14 and 16 seed structures; we stopped sampling when the physical meaning of the first 3 tICs (see Results) stabilized. The final data set was thus composed of 59 trajectories, each of 2.8 μs. This constitutes an aggregate sampling of 0.16 ms. IVAC was run again on the entire data set of ∼16 million time points as the starting point for further analysis.

### Clustering, MSM, and PCCA

Each tIC corresponds to a slowly decorrelating mode, and its associated eigenvalue corresponds to a timescale. We observed a spectral gap after the first six tICs (Figure S12a) and thus used those coordinates as the basis for the construction of a Markov state model (MSM).^30,31^ Specifically, we clustered the structures into 1000 microstates using the *k*-means algorithm in PyEMMA 2.5.7.^50^ Using a lag time of 100 ns, we computed the transition matrix between the microstates. The top eigenvectors of the transition matrix were then used to further group the microstates into 10 clusters, using Perron cluster analysis (PCCA).^34^ The MSM was validated as shown in Figure S12b,c.

To test the robustness of the analysis, we projected the centers of the obtained clusters on other IVAC spaces, using different lag-time ranges (1–20, 1–200, and 1–500 ns) and input basis sets (using 12, 15, and all α-carbons excluding neighbors closer than 4 residues). We found that the first 3 tICs separate the main 5 clusters in a consistent manner: tIC1 separates clusters 0 and 1, tIC2 separates clusters 0 and 3, and tIC3 separates clusters 2 and 4. Use of a coarser discretization for the MSM construction (500 microstates) and longer MSM lag times (200 ns) yielded similar clusters.

### Estimating Protection Factors from the Structural Ensemble

We estimate protection factors for each H_N_ site *i* (*f*_*i*_) based on the number of hydrogen bonds that each backbone amide forms with an acceptor atom in the protein chain (HB_*i*_^p^) and the number of hydrogen bonds it forms with any water oxygen (HB_*i*_^w^). We use four different models that combine these two quantities in different manners to ensure our results are not specific to a particular model. We refer to the models as Park, Ratio, Difference, and Bound-Difference. They all rely on the idea that an amide site is more protected the more hydrogen bonds it forms with other protein residues and the fewer with water.

The Park model uses the definition proposed by Park et al.^51^
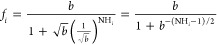
1where NH_*i*_ is defined as
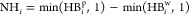
2The base parameter *b* is an arbitrary constant. Park et al. use values between 10^4^ and 10^6^, but we set it to 500 to make the maximum ∼250, close to the maximum experimental values in ref (15).

The Ratio model is
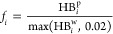
3where the max function prevents division by zero for structures with HB_*i*_^w^ = 0.

The Difference model is
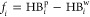
4Finally, the Bound-Difference model is
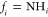
5where NH_*i*_ has the same form as in eq 2. Note that in this case, *f*_*i*_ can only take values of 1, 0, or −1.

To compute HB_*i*_^p^ and HB_*i*_^w^, we tested several donor–acceptor distance cutoffs (0.35, 0.40, 0.50, and 0.60 nm) and hydrogen-donor–acceptor angle cutoffs (40, 50, 70, and 90°). The mean PF for each H_N_ site *i* for each cluster *j*, PF_*ij*_, was computed by averaging over 4000 sample structures within it. The ensemble protection factor for one site, PF_*i*_, is taken as the weighted average across clusters 0–4.

In general, our results are consistent across model and parameter space. Individual absolute values of PF_*i*_ change depending on both the model and the angle-distance cutoffs used to define the hydrogen bonds, but consistent trends are observed (Figure S9). The correlations with experimental values ranged from 0.59 to 0.82, as shown in Figure S10. We observe that more restrictive angle and distance cutoffs tend to favor counting protein–protein hydrogen bonds, which results in higher protection factors. The correlations are particularly low when a loose angle cutoff is combined with a very restrictive distance requirement because hydrogen bonds of an H_N_ with the side chain of its residue are counted, while those with water are neglected.

## Results

Our goal is to characterize the diversity of structures populated by human insulin under low pH conditions (i.e., with protonated titratable residues), which are commonly used in NMR experiments. To ensure that we adequately sampled the conformational space available to the system, we employed the pipeline in Figure 2, as detailed in Materials and Methods. In brief, we used 15 structures from 6 μs of parallel tempering,^28,29^ with temperatures ranging from 303 to 415 K to seed room temperature (303.15 K) unbiased molecular dynamics simulations, each of which was 2.8 μs. Based on the projection of these data onto the first three time-lagged independent components (tICs),^32,33^ we iteratively expanded this data set by seeding additional trajectories until physical interpretations of the first three tICs (discussed below) stopped changing. The final data set had 59 trajectories totaling 165.2 μs of unbiased simulation, with structures saved every 10 ps for more than 1.6 million structures in total. We projected the structures onto the first six tICs and clustered them into 1000 states using the *k*-means algorithm. We then constructed a Markov state model^30,31^ (MSM) and used the PCCA algorithm^34^ to group the MSM microstates into 10 clusters based on the MSM transition matrix. We label the resulting clusters 0–9 in order of decreasing population. The analysis that we present is based on these 10 clusters, which range in population from 38.7 to 1.5% (see Table S1) and more specifically on the five major ones with populations greater than 9%, which account for ∼85% of the total population.

### Structural Features of the Clusters

**Figure 3:**
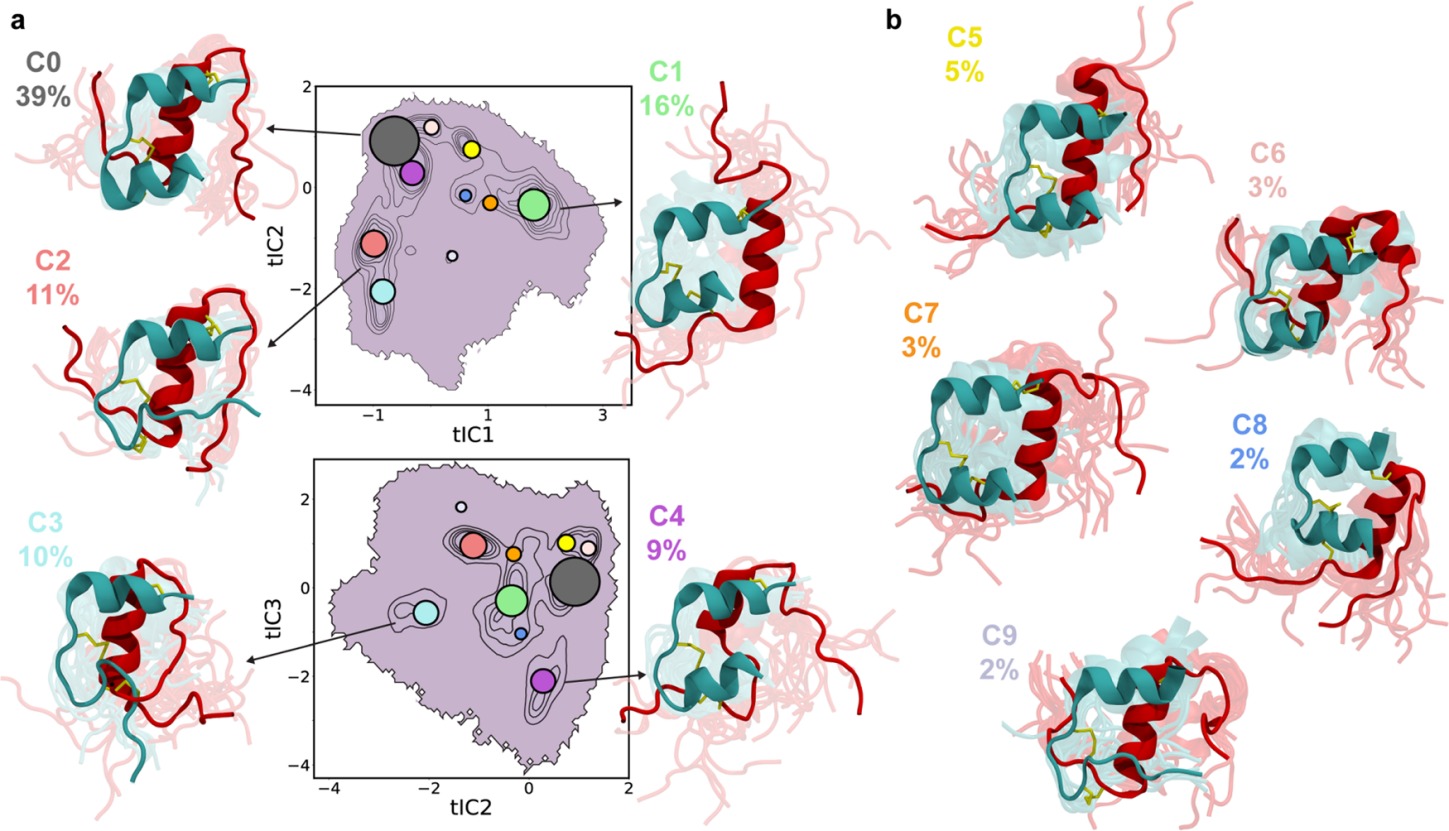
(a) Projection of all sampled structures onto tIC1 vs tIC2 (top) and tIC2 vs tIC3 (bottom), with clusters indicated. Each circle is centered on the median tIC values for a cluster, and the radius is proportional to the physical weight of the cluster. Representative structures for clusters 0–4, constituting ∼85% of the population, are shown. The A-chain is teal, and the B-chain is red. (b) Clusters 5–9, constituting the remaining 15% of the population.

**Figure 4:**
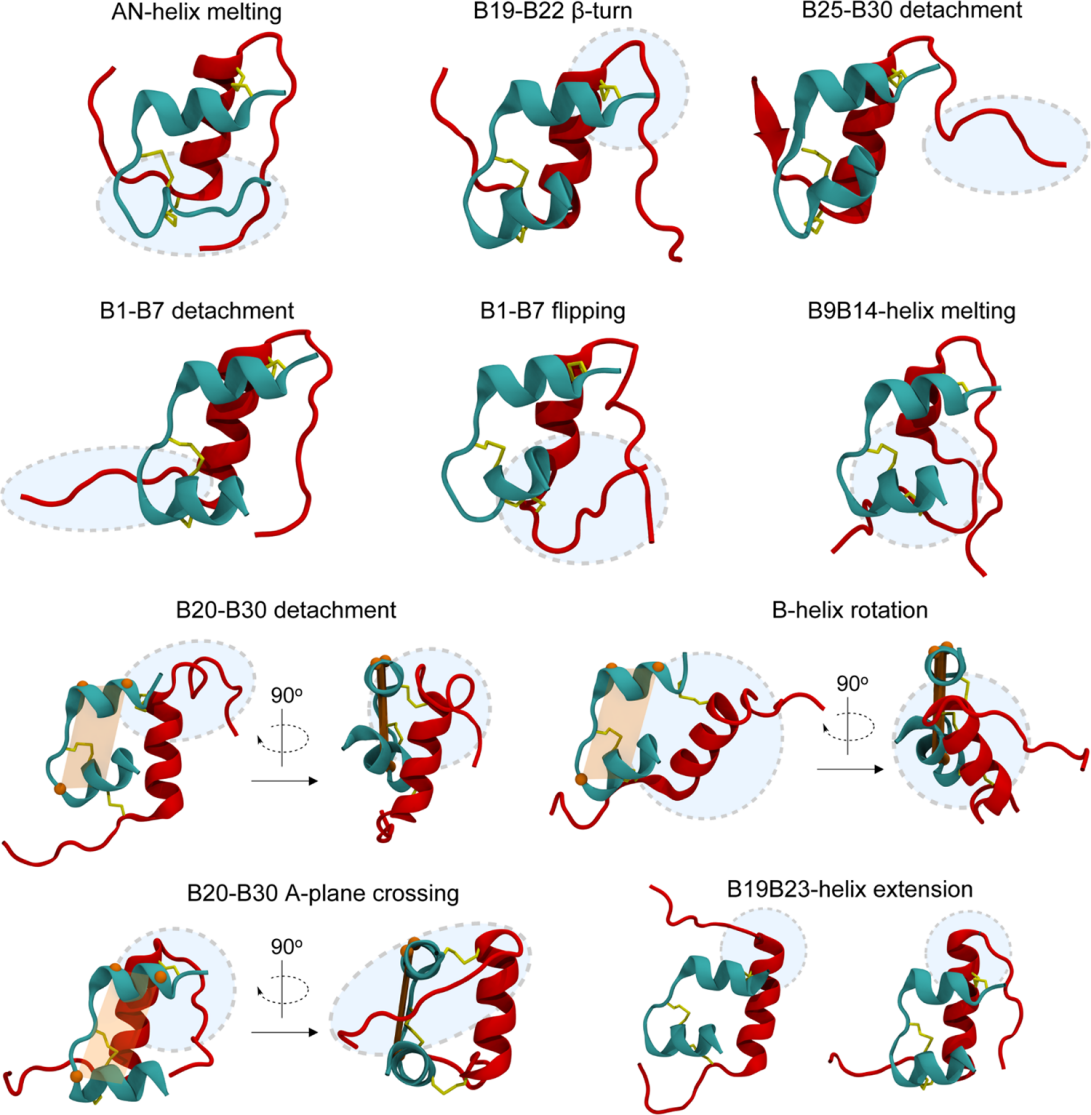
Structures illustrating the elements of disorder that we observe in clusters 0–9, which are summarized in Table 1. Further details regarding conformational heterogeneity of the B19–B23 segment are shown in Figure S3. B-helix rotation and B20–B30 A-plane crossing are defined with respect to the A-plane, which contains the C_α_ atoms of A9, A13, and A20. The three atoms and the A-plane are shown in orange in the relevant panels, as well as in the B20–B30 detachment panel for comparison. Two different structures are shown for B19–B23-helix extension, corresponding to the structures seen in clusters 1 (left) and 5 (right).

Representative structures from the 10 clusters are illustrated in Figure 3. The AC-helix and the Leu^B15^-Cys^B19^ segment of the B-helix are well-folded in all clusters. Otherwise, the clusters differ, with the conformational heterogeneity taking three main forms: AN-helix melting, detachment of the B1–B7 segment, and detachment of the B20–B30 segment. Each such element is generally present or absent in all of the structures in a given cluster. Below, we describe the clusters in more detail, including additional elements that are present in only a fraction of the structures within a cluster. An example is the melting of the Ser^B9^-Ala^B14^ region of the B-helix, which is present in only 51% of structures in cluster 4. These results are summarized in Table 1, and example structures are shown in Figures 4 and S3. The collective variables used to determine the presence of the elements are described in the Supporting Information and shown in Figure S4.

**Table 1:**
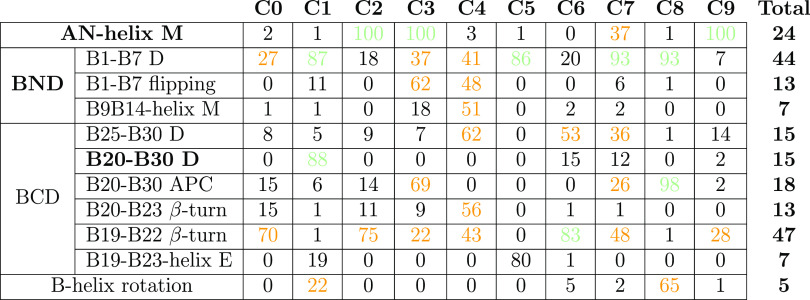
Summary of the Observed Structural Elements in Clusters 0–9 (C0–C9), Expressed as the Physically Weighted Percentage of Structures Presenting a Given Element of Disorder within Each Cluster^a^

a Green indicates that 80% or more of structures within a cluster contain a given element, while orange indicates 20–80%. Total percentages in the ensemble are listed in the last column. Structural elements that correlate with the first three tICs are shown in bold. Abbreviations: BND, B-chain N-terminus detachment; BCD, B-chain C-terminus detachment; D, detachment; M, melting; E, extension; APC, A-plane crossing.

**Cluster 0** represents 38.7% of the population. It contains structures consistent with the T state, though some exhibit detachment of B1–B7 (27%). We find that in most structures of cluster 0 (70%), the β-turn shifts from B20–B23 (as in the T state) to Cys^B19^-Arg^B22^, and the B23 H_N_···O=C B20 hydrogen bond is replaced by a B22 H_N_···O=C B19 hydrogen bond (Figure S3 Top and Middle). The ϕ dihedral angle of Gly^B23^ accordingly changes from ∼80 to ∼−70° (Figure S4).

**Cluster 1** represents 16.0% of the population and contains structures in which the B-chain residues after B20 are detached, disrupting the B20–B23 β-turn. The detached B-chain C-terminus can take many conformations and can even align with the AC-helix (Figure 3a). Briefly, 19% of the structures in cluster 1 exhibit an extension of the B-helix up to Gly^B23^ (B19–B23-helix extension). In 87% of the structures of cluster 1, the B-chain N-terminus detaches at B7, breaking native inter-chain contacts between the B1–B7 and the A6−A11 segments, as described below. In 22% of the structures in cluster 1, the B-helix rotates with respect to the A-chain (Figure 4, B-helix rotation). In particular, the top part of the B-helix rotates outward from the plane containing the C_α_ atoms of A9, A13, and A20, which we call the A-plane. These atoms and the A-plane are shown in orange in the B20–B30 detachment and B-helix rotation panels of Figure 4. An inspection of the trajectories reveals that structures with and without this rotation do not readily interconvert. Clustering the MSM into 12 states instead of 10 splits cluster 1 into two clusters, one with the rotation and one without (1a and 1b, respectively, in Figure S1).

**Cluster 2** represents 11.0% of the population and contains structures in which the AN-helix is melted while the B-chain is mostly native-like. Similar to cluster 0, the β-turn predominantly shifts to B19–B22 (75%).

**Cluster 3** represents 9.6% of the population and combines a melting of the AN-helix (as in cluster 2) with either partial or complete B-chain N-terminus detachment. Partial detachment (37%) leads to B1–B7 conformations similar to those in cluster 1. Total detachment (62%) allows Phe^B1^-His^B5^ to go around the B-helix and form non-native hydrophobic contacts with Val^B12^ and Tyr^B16^. 69% of the structures in cluster 3 present detachment of the B20–B30 segment toward the AC-helix, i.e., outward from the previously defined A-plane (Figure 4, B20–B30 A-plane crossing). This A-plane crossing (APC) breaks the B20–B23 β-turn and changes the Glu^B21^ ϕ dihedral angle (Figure S4). A non-native hydrogen bond, B21 H_N_···O=C B16, is also formed (Figure S3 Bottom). This feature is present in clusters 0 and 2 as well but seems to be facilitated by the combined melting of the AN-helix and the flip of the B1–B7 segment (Figure S6).

**Cluster 4** represents 9.4% of the population and contains structures in which the N-terminus of the B-chain is partially (48%) or wholly (51%) detached. It also presents considerable disorder in the Phe^B25^-Ala^B30^ segment (62%), and among the major clusters is the one where the B20–B23 β-turn is more prevalent (56%). In approximately half of cluster 4, the B-helix is melted between Gly^B9^ and Ala^B14^; such structures are rarely found in other clusters, except a small fraction of those in cluster 3. Nevertheless, they are consistent with the observation that serine substitution at B8 impacts not only B1–B7 but also NOEs among B9–B14.^52^

We summarize the clusters with populations below 5% (Figure 3b) as follows. **Cluster 5** (4.7%) contains structures in which the B-helix extends to B23, but, unlike cluster 1, with the rest of the B-chain C-terminus packed against the B-helix (see Figure 4 B19–B23-helix extension, right). **Cluster 6** (3.5%) contains structures with Arg^B22^-Thr^B27^ folded into a separate α-helix that packs against the B-helix; in other structures, the B-helix extends to B25. In structures in **cluster 7** (3.4%), the B-chain N-terminus is partially detached, and the B20–B23 β-turn is disrupted. The remainder of the B-chain C-terminus is mostly attached. **Cluster 8** (2.2%) contains structures with a partial detachment of the B-chain N-terminus and rotation of the B-helix across the A-plane. A-plane crossing of the B20–B30 segment is present in 98% of cluster 8, facilitated by this same rotation. Finally, **cluster 9** (1.5%) combines melting of the AN-helix (as in cluster 2) with loss of the B20–B23 β-turn and partial detachment of the B-chain C-terminus.

### Physical Interpretation of tICs

Since tICs characterize the slowest collective motions in the system, we expect the leading tICs to distinguish the conformational changes separating major clusters. Consistent with this idea, we see that tIC1 separates cluster 0 (native-like) from cluster 1 (B20–B30 detachment); tIC2 separates cluster 0 from cluster 2 (melting of the AN-helix) and cluster 3 (melting of the AN-helix and detachment of the B1–B7 segment); tIC3 separates cluster 2 from cluster 4 (B1–B7 detachment). Based on these, we tentatively associated tIC1 with detachment of the B-chain C-terminus, tIC2 with melting of the AN-helix, and tIC3 with detachment of the B-chain N-terminus around B7 (see Figure S1 for tIC4). To confirm these relationships, we characterized the distances between the C_α_ atoms, hydrogen bonds, secondary structure content, and contact numbers. Multiple measures were highly correlated (Pearson’s |*R*| ≥ 0.8) with each of the first three tICs (Figure S2), which confirmed our hypotheses.

### Kinetics

Both the MSM and the IVAC analysis that produce the tICs yield timescales of relaxation within the population. Although the relative timescales of different modes of relaxation are consistent across methods, we find the absolute timescales differ by up to an order of magnitude and are sensitive to algorithmic choices such as the number of states included. We thus report ranges for the timescales associated with the main elements of disorder: 2.0–20.4 μs for B20–B30 detachment, 0.9–7.3 μs for AN-helix melting, and 0.6–6.0 μs for B1–B7 detachment. These timescales are of the same order as ones for hairpin folding (1–20 μs) and helix formation (1–2 μs).^53^

**Figure 5:**
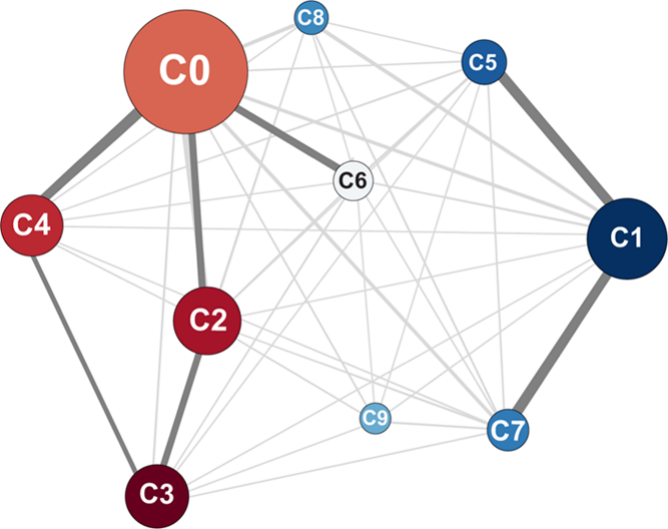
Network plot illustrating population fluxes between clusters 0 and 9. The node radii are proportional to the clusters’ equilibrium populations and are positioned using the ForceAtlas algorithm^54^ such that states which exchange rapidly are closer to each other. Edge thicknesses are proportional to the fluxes between nodes and fluxes over 25% of the maximum flux, which is between clusters 0 and 4, are highlighted. The nodes are colored according to the cluster mean value of the first nontrivial eigenvector of the MSM transition matrix.

One way of visualizing the relaxation of the population is through a network plot in which the thicknesses of the edges are proportional to the fluxes between clusters at equilibrium (Figure 5). This shows that there are two subnetworks between which exchange is slower (the first timescale range above) and within which exchange is faster (the other two timescale ranges above). One subnetwork is composed of clusters 0, 2, 3, 4, and 6, and the other subnetwork is composed of clusters 1, 5, and 7. The fluxes between clusters 0, 2, 3, and 4 indicate that exchange from cluster 0 (native-like) to cluster 3 (with AN-helix melting and B1–B7 detachment) proceeds stepwise. Either the B-chain N-terminus first detaches and then the AN-helix melts (C0 → C4 → C3), or the other way around (C0 → C2 → C3). The dynamics within the subnetwork containing clusters 1, 5, and 7 are less straightforward to interpret but appear to involve rearrangements of the B20–B30 segment, including the β-turn.

### Connection to Hydrogen-Exchange Measurements

To validate the structural ensemble and illustrate its utility in interpreting experimental measurements, we used the structures to estimate hydrogen–deuterium exchange protection factors for amide hydrogen (H_N_) sites. The classical model of exchange assumes that exchange occurs after a site undergoes a transition from a protected (closed) to a solvent-exposed (open) state^55,56^
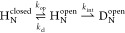
6where *k*_op_ and *k*_cl_ are the opening and closing rates, respectively, and *k*_int_ is the intrinsic rate of exchange for a given residue. Using either NMR or mass spectrometry, experiments measure the apparent rate at which protons exchange (*k*_obs_), which can be expressed as
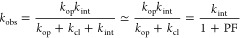
7where the so-called Protection Factor (PF) is defined as PF = *k*_cl_/*k*_op_. The approximation made in eq 7 is that *k*_cl_ ≫ *k*_int_, which is known as the EX2 regime.^57^ This is equivalent to saying that the site undergoes many opening and closing events before the exchange, which is consistent with the microsecond-timescale kinetics discussed in the previous section. Since residue intrinsic rates are available from measurements on model peptides, *k*_obs_ measurements can be used to obtain PF for each H_N_ site.

Although in principle, the equilibrium between the closed and open states of each site is available from molecular dynamics simulations, in practice, it is not straightforward to define the closed and open states. Qualitatively, they are expected to depend on the site’s exposure to the solvent, as well as its hydrogen bonds.^57,58^ Existing models for estimating protection factors incorporate these ideas in ad hoc ways (see refs (51, 59) for reviews and comparisons of models). We tested several such models, as described in Materials and Methods. The results we report in the main text are for the model that yielded the best overall agreement with the available data, but in general, the trends that we observe are consistent across models. We compare the simulated PFs to measurements for KP-insulin,^15^ which is predominantly monomeric in solution.

**Figure 6:**
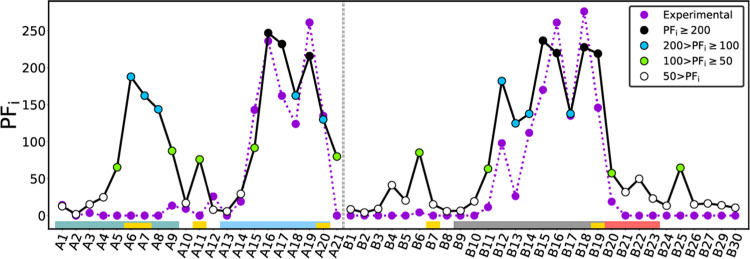
Ensemble protection factor PF_*i*_ for each H_N_ site *i*, taken as the weighted average for clusters 0 to 4. Different colors are used depending on the computed PF (see legend). Experimental protection factors come from Hua et al.^15^ Results shown are for the Park model,^51^ with hydrogen-bond hydrogen-donor–acceptor angle and donor–acceptor distance cutoffs of 70° and 0.5 nm, respectively. Secondary structure motifs of insulin are indicated in the lower part as colored bars, following the color code in Figure 1.

Overall, the protection factors computed for the ensemble (Figure 6) are in reasonable agreement with the measurements (Pearson’s *R* = 0.82). In both simulation and experiment, the largest PFs occur around A16–A19 and B15–B19. These sites are located in the AC-helix and the part of the B-helix that is well-folded throughout our simulations (Figure S8). Experimentally, Hua et al.^15^ found the rest of the sites to have relatively low protection factors. In our case, we see a similar trend but overestimate the protection factors of A5−A9, A11, A21, B4, B6, B11, B13, B22, and B25. Notably, we compute intermediate to high protection factors for A5−A9 within the AN-helix. This is due to cluster 0, which is native-like and dominates averages due to its large population.

### Protection Factors of Individual Clusters

**Figure 7:**
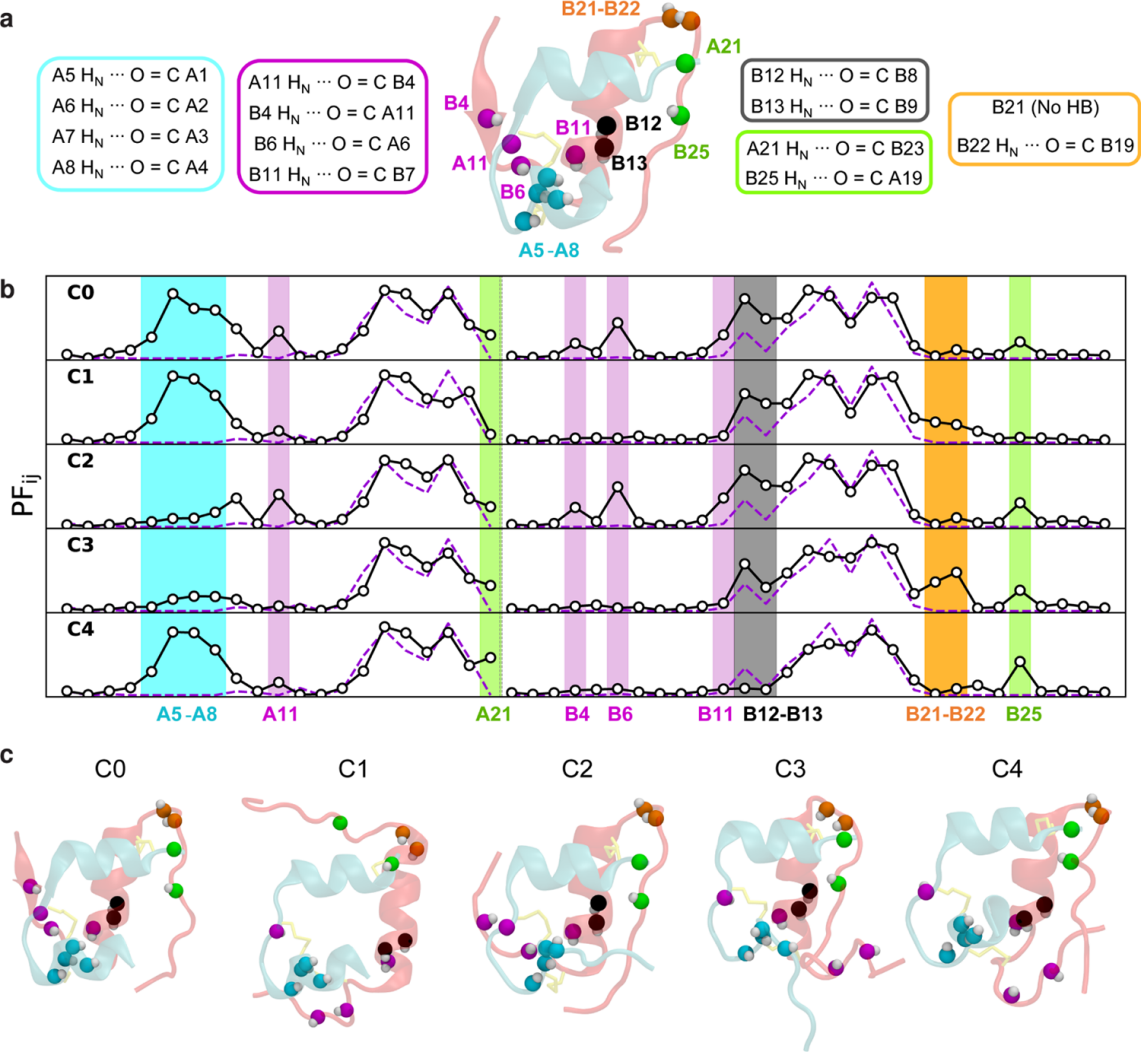
Mean protection factor for individual clusters 0 to 4 and representative structure of each cluster. H_N_ sites presenting a coefficient of variation in PF_*i*_ of greater than or equal to 10% are depicted in the structures using various colors for N atoms and white for H atoms. (a) Native structure resulting from minimization and solvation of the NMR structure (PDB ID 2JV1^11^). H_N_ sites of interest are represented, and their native hydrogen-bond state is listed.^25^ (b) Mean simulated protection factor (white circles) for clusters 0 to 4. Experimental values are plotted as a purple dashed line. Sites of interest are highlighted following the code in panel a. (c) Representative structures for clusters 0 to 4.

From cluster to cluster, the simulated protection factors of some sites vary significantly (Figure S11). Specifically, sites with variances that are at least 10 times their means are shown in Figure 7a. These sites correspond closely to the largest deviations between the computed protection factors and experimental values. Hua et al.^15^ noted the surprisingly low protection factors of most of these sites (plus B23, which is consistently exposed in our data set) given that they appear to form protein–protein hydrogen bonds in the T state. They attributed their findings to local conformational fluctuations, such as detachment of the B-chain N-terminus or melting of the AN-helix, which is consistent with the structures that we observe.

In this section, we discuss how the exposure of the sites with highly variable PFs relates to the conformational heterogeneity that we observe. We find that there are five groups of correlated sites, as depicted in Figure 7 in various colors, and we organize our discussion around them. Results using a different model are shown in Figure S9b.

1. The sites in **cyan** (A5–A8) are in the AN-helix. Accordingly, they are highly protected in clusters 0, 1, and 4 (each with a largely conserved AN-helix), and exposed in clusters 2 and 3 (each with a predominantly melted AN-helix).
2. The sites in **magenta** (A11, B4, B6, and B11) are exposed by detachment of the B-chain N-terminus. They are protected in clusters 0 and 2, which maintain native inter-chain (e.g., A11 H_N_ ··· O=C B4) and intra-chain (e.g., B11 H_N_ ··· O=C B7) hydrogen bonds, but are unprotected in clusters 1, 3, and 4, which break these hydrogen bonds upon B-chain N-terminus detachment.
3. The sites in **black** (B12 and B13) are in the B-helix. Protected in almost all of the observed clusters, these sites are exposed by the partial melting of the B-helix, a feature unique to cluster 4.
4. The sites in **green** (A21 and B25) are exposed by the detachment of the B20–B30 segment. Moderately protected in clusters 0, 2, 3, and 4, in which B20–B25 remain close to the protein core forming inter-chain hydrogen bonds, they are unprotected in cluster 1, in which B20–B30 detachment is most prevalent.
5. The sites in **orange** (B21–B22) are in the β-turn. These sites are solvent-exposed in clusters 0, 2, and 4, in which the β-turn shifts to B19–B22 (Figure S3). These sites are moderately protected in cluster 1, owing to the extension of the B-helix to B23, and in cluster 3, owing to B20–B30 A-plane crossing, which results in a B21 H_N_ ··· O=C B16 hydrogen bond and burial of B22.

## Discussion

Here, we used all-atom molecular dynamics simulations and enhanced sampling methods together with kinetic clustering of conformational substates to provide a microscopic view of insulin’s structural ensemble in solution. Our study reveals various forms of conformational variation and a higher degree of disorder than indicated in prior structural studies. The most prominent elements of disorder are AN-helix melting, B-chain N-terminus detachment, and B-chain C-terminus detachment. These elements are associated with microsecond-scale exchange kinetics between the clusters. The B-chain is further characterized by a diversity of conformations at B1–B7, melting of the B-helix at B9–B14, and diversity of conformations at B19–B23, which contains the β-turn that governs B-chain hinging. Although we model insulin at low pH because those solution conditions are used experimentally to stabilize the monomer, we expect the disorder that we observe to be present at neutral pH as well.^60^

We were able to observe the extensive disorder in the structural ensemble owing to the enhanced sampling procedures that we used, which combined parallel tempering with selectively seeding relatively long (2.8 μs) unbiased simulations based on the tICs for an aggregate sampling of 165 μs. Our results are consistent with those of Zoete et al.,^25^ who performed two unbiased simulations of 5–10 ns of the porcine insulin monomer starting from crystallographic T-state structures. The disorder they reported at the B1–B7 and B25–B30 segments, as well as the shift of the B20–B23 β-turn to B19–B22, is encompassed in the structures we observe in native-like cluster 0, consistent with the short duration of their simulations compared with the microsecond timescales we estimate. The conformations in the other nine clusters have not been observed in previous simulation studies.

By simulating protection factors, we show that our results are consistent overall with previous hydrogen-exchange measurements.^15^ Although the ensemble average overestimates the protection of certain H_N_ sites, individual clusters provide microscopic structures that are consistent with experimental data. They thus provide support for the hypothesis that conformational heterogeneity in specific regions is the origin of the unexpectedly low protection of these sites.^15^ At the same time, the fact that we overestimate the protection of these sites suggests that we underestimate the extent of disorder in the ensemble. This may reflect issues with the force field (e.g., helix over-stabilization^61,62^), sampling, and the model for estimating protection factors from the structures. The specific insulin sequences are also different (wild-type human insulin versus KP-insulin), and it is possible that the sequence substitutions (Pro^B28^ → Lys^B29^ and Lys^B29^ → Pro^B28^), which are in the B-chain C-terminus, destabilize the nearby AN-helix. That said, our work underscores the importance of sampling key unfolded (open) states for PF computation; indeed, the consistency of our results across different models for estimating PFs from the structures suggests the structural ensemble may have more impact than the choice of model.

Intrinsically disordered regions of proteins are particularly challenging to characterize experimentally, and thus there are limited avenues to compare our ensemble to solution-phase experimental data.^63^ Hydrogen-exchange measurements are made on timescales of minutes to hours, which is orders of magnitude slower than the interconversion between states. Indeed all NMR techniques that employ chemical shift measurements are motionally averaged over the timescale of the measurement, typically 0.1–10 s. One approach that should prove useful for distinguishing rapidly interconverting protein conformations is amide I infrared spectroscopy of specifically labeled backbone carbonyls. The frequency and lineshape of the carbonyl resonance report on hydrogen bonding to the carbonyl and water exposure with picosecond time resolution.^64,65^ Used in conjunction with simulation,^66^ it has been used to characterize structural disorder and dynamics in several systems,^67,68^ including insulin.^69−72^

While our study does not provide information about the mechanisms through which insulin binds the receptor, it shows that the B-chain N- and C-termini readily detach. Single-particle cryo-electron microscopy (cryo-EM) structures of the insulin-receptor complex,^4,5^ as well as photo-crosslinking experiments,^73,74^ show that the B-chain C-terminus must detach from the hydrophobic core to bind the receptor, and sequence changes that decrease^75−77^ or increase^78−80^ the flexibility of this segment result in lower and higher biological activity, respectively. Although the need for the B-chain N-terminus to reorient for insulin to bind the receptor is not apparent from the structures of the complex,^4,5^ substitutions at Gly^B8^ indicate that binding relies on the B8 ϕ dihedral angle accessing values characteristic of those we observe in structures with B1–B7 detachment,^52,81,82^ suggesting the functional importance of this element of disorder as well.

On the other hand, cluster 3 is consistent with NMR structures of wild-type human insulin under amyloidogenic conditions (60 °C, pH 2.4),^83^ which exhibit disorder in the form of AN-helix melting and B1–B7 detachment. The NMR structures are relatively well-structured at the B-chain C-terminus, though a number of them have B21 ϕ dihedral angle values that are consistent with those observed for cluster 3 (Figure S4). Substitutions at Ile^A2^, Val^A3^, and Thr^A8^ that are expected to favor helix formation reduce thermodynamic stability but increase resistance to fibrillation. Given that it is thought that A2 and A3 are conserved because they make receptor contacts,^83^ we speculate that the amount of disorder we observe in our ensemble reflects an evolutionary trade-off between the promotion of receptor binding and fibrillation.

The structures that we observe can serve as starting points for the rational design of insulin analogues. The recent introduction of a thermostable active monomeric single-chain insulin analogue in which the B-chain C-terminus is linked to the A-chain N-terminus via a six-residue peptide^84^ shows that there is wide scope for new therapeutics. NMR structures of this complex suggest that a linker of sufficient length is needed to enable conformational change upon receptor binding, pointing to the importance of considering the accessible conformational space in the design. The interplay of B1–B7 with B9–B14 and the heterogeneity at B19–B23, which have received less attention than the AN-helix and B-chain C-terminus, may be worth further consideration for design. The broader question of how to think of intrinsic disorder in molecular association remains open.

## Supporting Information Available

Additional methods and Supporting Information Figures (S1–S12, as referenced in the text), as well as coordinate files for 10 sample structures for each cluster (0–9) and 10 sample structures for each microstate in the MSM (PDF)
