# Supplementary material for: Structural Ensemble of the Insulin Monomer
Source: Biochemistry. 2021 Oct 12;60(42):3125–36. doi: 10.1021/acs.biochem.1c00583 (PMC8552439; doi:10.1021/acs.biochem.1c00583)
Supplement: Supplementary file 1 — bi1c00583_si_003.pdf [file bi1c00583_si_003.pdf]

Structural ensemble of the insulin monomer

Luis Busto-Moner,[†] Chi-Jui Feng,[†] Adam Antoszewski,[†] Andrei Tokmakoff,[*,†,‡,¶]
and Aaron R. Dinner[*,†,‡,¶]

†Department of Chemistry, The University of Chicago, Chicago, Illinois 60637, United States

‡James Franck Institute, The University of Chicago, Chicago, Illinois 60637, United States

¶Institute for Biophysical Dynamics, The University of Chicago, Chicago, Illinois 60637, United States

E-mail: tokmakoff@uchicago.edu; dinner@uchicago.edu

Characterization of disorder in clusters 0 to 9. Here, we detail the collective variables and cutoffs that we use to assess the presence or absence of structural elements (e.g., for the percentages shown in Table 1). How the values of these collective variables are distributed within each of the major clusters is shown in Figure S4. All angles range from –180 to 180°.

- **AN-helix melting**: Melting of the AN-helix is characterized by the degree of helicity of the A1-A9 segment, $\mathrm{H_{A1A9}}$. This is measured as implemented in PLUMED 2.5.6,[1–4] using the RMSD, r_i, with respect to a an idealized helix model as follows:

$$\mathrm{H_{segment}} = \sum_{\mathrm{i}} \frac{1 - (\mathrm{r_i}/0.08)^8}{1 - (\mathrm{r_i}/0.08)^{12}}. \tag{1}$$

  The sum runs over every segment of six contiguous residues along the sequence of interest. There are four such segments for A1-A9, so $\mathrm{H_{A1A9}}$ ranges from 0 (totally melted) to 4 (totally folded). We consider the AN-helix melted if $\mathrm{H_{A1A9}} < 2$.

- **B1-B7 flipping**: Flipping of the B1-B7 segment across the B-helix is measured with the pseudo-dihedral angle formed by the C_α atoms of B3, A15, B18, and B15 (Ψ_{B1B7f}; Fig. S5). We count structures as having flipping if $\Psi_{\mathrm{B1B7f}} > 10°$.

- **B1-B7 detachment**: Partial detachment of the B1-B7 segment is measured with the angle formed by the C_α atoms of B2, B9, and B20 (Ψ_{B1B7d}; Fig. S5). Only structures that do not show B1-B7 flipping are considered. We count structures as having B1-B7 detachment if $\Psi_{\mathrm{B1B7d}} > 85°$.

- **B9-B14 melting**: We characterize the degree of helicity of the B9-B14 segment using Eq. 1. We consider the B-helix melted if $H_{\mathrm{B9B14}} < 5$.

- **B20-B30 detachment**: The detachment of the B20-B30 segment is measured with the pseudo-dihedral angle formed by the C_α atoms of A13, A18, B19, and B25 (Ψ_{B20B30d}; Fig. S5). We count structures as having B20-B30 detachment if $\Psi_{\mathrm{B20B30d}} < 0°$. We do not count any structures in cluster 5 because extension of the B-helix to B23 also makes $\Psi_{\mathrm{B20B30d}} < 0°$ even without B20-B30 detachment.

- **B20-B30 APC**: A-plane crossing by the B20-B30 segment (Fig. 4) is quantified with the ϕ dihedral angle of B21 ($\phi_{\mathrm{B21}} > 0°$), which flips 180° as B21 forms a hydrogen bond with B16 (Figure S3). Only structures without B20-B30 detachment are considered.

- **B25-B30 detachment**: The detachment of the B25-B30 segment is measured with the angle formed by the C_α atoms of B16, B20, and B27 (Ψ_{B25B30d}; Fig. S5). We consider B25-B30 detachment if $\Psi_{\mathrm{B25B30d}} < 50°$. Only structures without B20-B30 detachment or B20-B30 APC are considered.

- **B20-B23 β-turn**: The Presence of the native β-turn is detected using the distance between the C_α atoms of B20 and B23 ($d_{\mathrm{B20B23}} < 0.56$ nm), as well as the ϕ dihedral angle of B23 ($\phi_{\mathrm{B23}} > 0°$). Only structures without B20-B30 detachment or B20-B30 APC rotation are considered.

- **B19-B22 β-turn**: In structures that do not show B20-B30 detachment or B20-B30 APC rotation, we detect the B19-B22 β-turn using $\phi_{\mathrm{B23}} < 0°$.

- **B19-B23-helix extension**: Extension of the B-helix up to B23 is quantified using $\mathrm{H}_{\mathrm{B18B23}} > 0.1$ and $\mathrm{H}_{\mathrm{B22B27}} < 0.1$ to distinguish from folding of the B22-B27 segment into a separate α-helix, as in cluster 6.

- **B-helix rotation**: The rotation of the B-helix with respect to the A-plane is measured using the pseudo-dihedral formed by the C_α atoms of A11, A14, B19, and B16 ($0.3 > \Psi_{\mathrm{B-hrot}} > -0.25$. We do not consider clusters 0, 2, 3, and 4.

Correlations between elements of disorder. For each structure in our data set, we define an indicator function h_i for the presence or absence of a given element of disorder i from Table 1. We compute the correlation between two elements of disorder i and j as

$$R_{ij} = \frac{\langle \overline{h_i \cdot h_j} \rangle - \langle \overline{h_i} \rangle \cdot \langle \overline{h_j} \rangle}{s_i \cdot s_j}, \tag{2}$$

where s_i is the standard deviation for h_i.

Correlation coefficients for all pairs of elements of disorder in Table 1 can be found in Fig. S6. We find positive correlations to be consistent with the features observed for the main clusters 0 to 4. Namely, we find the highest correlations between elements in cluster 1 (B1-B7 detachment with B20-B30 detachment, 0.36; B25-B30 detachment with the B20-B23 β-turn, 0.33), then between elements in cluster 4 (B20-B30 detachment with the B-helix rotation, 0.26; B1-B7 flipping with B9B14-helix melting, 0.28), and then between elements in cluster 3 (B1-B7 flipping, AN-helix melting, and B20-B20 A-plane crossing rotation taken pairwise, between 0.23 and 0.26).

The strongest negative correlations are observed between elements of disorder involving the same residues in an incompatible form, such as the APC rotation of the B20-B30 segment and the B19-B22 β-turn (–0.44). By contrast, the detachment of the B20-B30 segment and

the melting of the AN-helix (–0.22) can physically occur at the same time, but they are rarely observed together.

Kinetics of exchange between clusters For a pair of clusters i and j, we compute the flux as:

$$f_{ij} = T_{ij} \cdot \pi_i, \tag{3}$$

where T_{ij} is the element of the coarse-grained cluster transition matrix $\mathbf{T}$ indicating the probability of exchange from i to j, and π_i is the stationary probability of the system to be in state i (as indicated in Table S1). Note that since $\mathbf{T}$ satisfies the detailed balance condition:

$$\pi_i \cdot T_{ij} = \pi_j \cdot T_{ji} \tag{4}$$

the forward and backward fluxes are equal.

References

(1) Bonomi, M., et al. Promoting Transparency and Reproducibility in Enhanced Molecular Simulations. *Nat. Methods* **2019**, *16*, 670–673.

(2) Tribello, G. A.; Bonomi, M.; Branduardi, D.; Camilloni, C.; Bussi, G. PLUMED 2: New Feathers for an Old Bird. *Comput. Phys. Commun.* **2014**, *185*, 604–613.

(3) Bonomi, M., et al. PLUMED: A Portable Plugin for Free-Energy Calculations with Molecular Dynamics. *Comput. Phys. Commun.* **2009**, *180*, 1961–1972.

(4) Pietrucci, F.; Laio, A. A Collective Variable for the Efficient Exploration of Protein Beta-Sheet Structures: Application to SH3 and GB1. *J. Chem. Theory Comput.* **2009**, *5*, 2197–2201.

(5) Hua, Q. X.; Jia, W.; Weiss, M. A. Conformational Dynamics of Insulin. *Frontiers in Endocrinology* **2011**, *2*, 48.

(6) Scherer, M. K.; Trendelkamp-Schroer, B.; Paul, F.; Pérez-Hernández, G.; Hoffmann, M.; Plattner, N.; Wehmeyer, C.; Prinz, J.-H.; Noé, F. PyEMMA 2: A Software Package for Estimation, Validation, and Analysis of Markov Models. *J. Chem. Theory Comput.* **2015**, *11*, 5525–5542.

Table S1: Cluster statistics.

**Cluster**	**Weight (%)**	**ΔG° (KJ/mol)**	**Structures (%)**	**Microstates (%)**
0	38.7	0.0	29.9	13.3
1	16.0	2.2	16.0	27.9
2	11.0	3.2	12.9	8.8
3	9.6	3.5	7.9	10.1
4	9.4	3.6	11.2	10.7
5	4.7	5.3	5.6	4.7
6	3.5	6.0	6.9	6.9
7	3.4	6.1	7.0	12.6
8	2.2	7.3	0.9	1.5
9	1.5	8.1	1.7	3.5

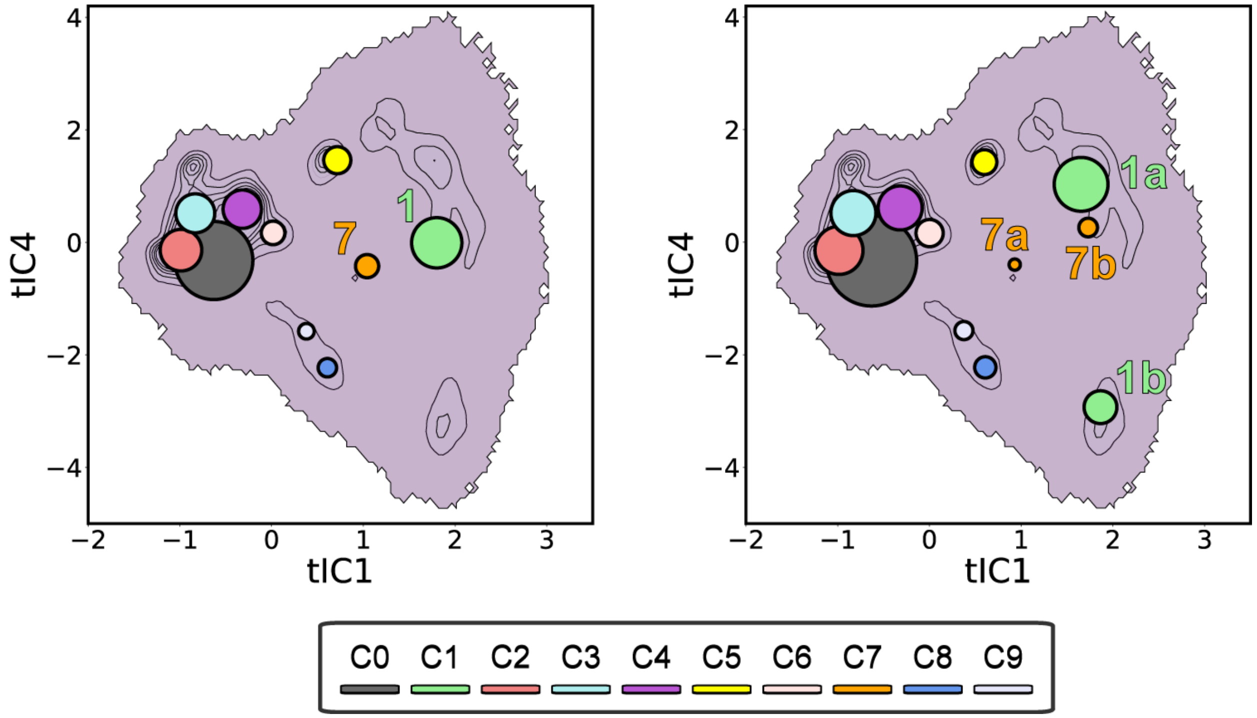

Figure S1: Projection of all sampled structures and clusters onto tIC1 versus tIC4 for 10 (left) and 12 metastable states (right). Each circle is centered around the median tIC values for each cluster, and the radius is proportional to the physical weight of the cluster. Based on the differences between clusters 1a and 1b, we interpret tIC4 as tracking the rotation of the B-helix such that it crosses the A-plane.

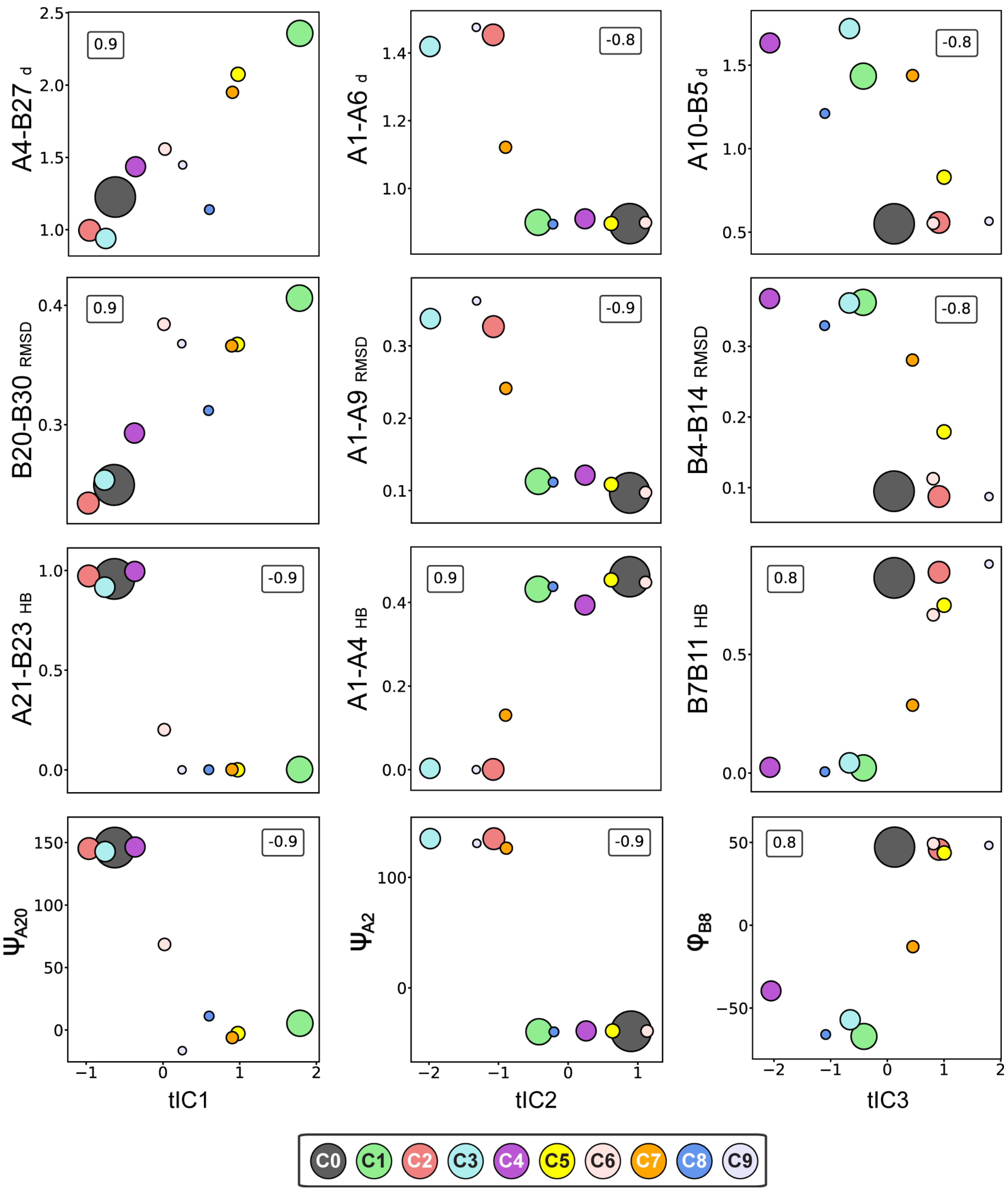

Figure S2: Scatter plots of clusters 0 to 9 along relevant collective variables vs tIC1 (left), tIC2 (middle) and tIC3 (right). Each circle is centered around the cluster weighted mean value for each CV, and the radius is proportional to the physical weight of the cluster. Pearson correlation coefficients between tICs and CVs are shown in upper-corner text boxes. First row ($X - Y_d$) shows distances between respective α-C of residues X and Y. Second row ($X - Y_{RMSD}$) shows backbone RMSD of atoms comprised between residue X and Y. Third row ($X - Y_{RMSD}$) shows the mean number of hydrogen bonds between residues X and Y. Fourth row (Ξ_X) includes angle Ξ (Φ or Ψ) of residue X.

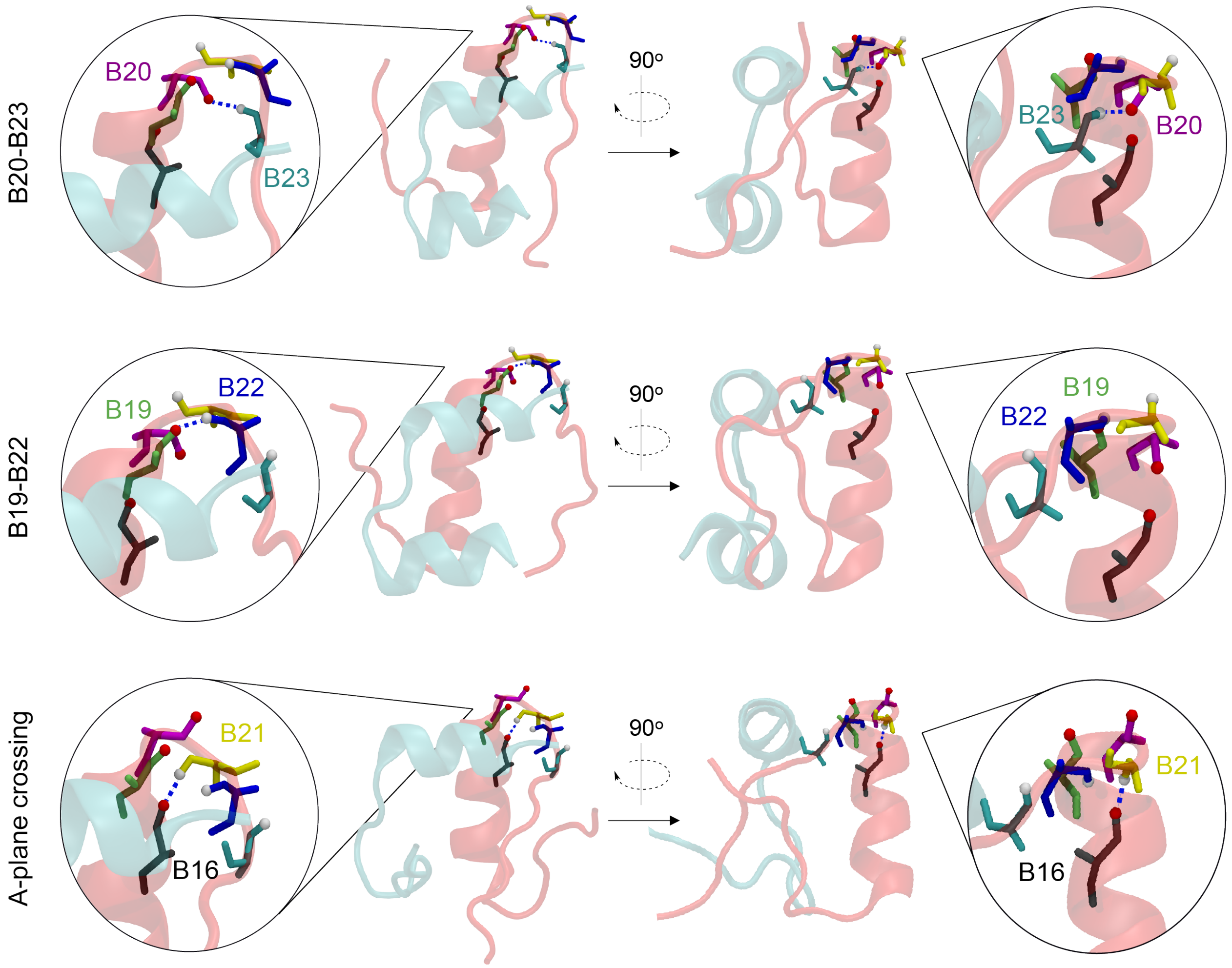

Figure S3: Main conformations of the B19-B23 segment: Gly^{B20}-Gly^{B23} β-turn (top), Cys^{B19}-Arg^{B22} β-turn (middle), and disrupted β-turn due to A-plane crossing detachment of the B20-B30 segment (bottom). Residues of interest are colored as follows: B16 (black), B19 (lime), B20 (magenta), B21 (yellow), B22 (blue), B23 (teal).

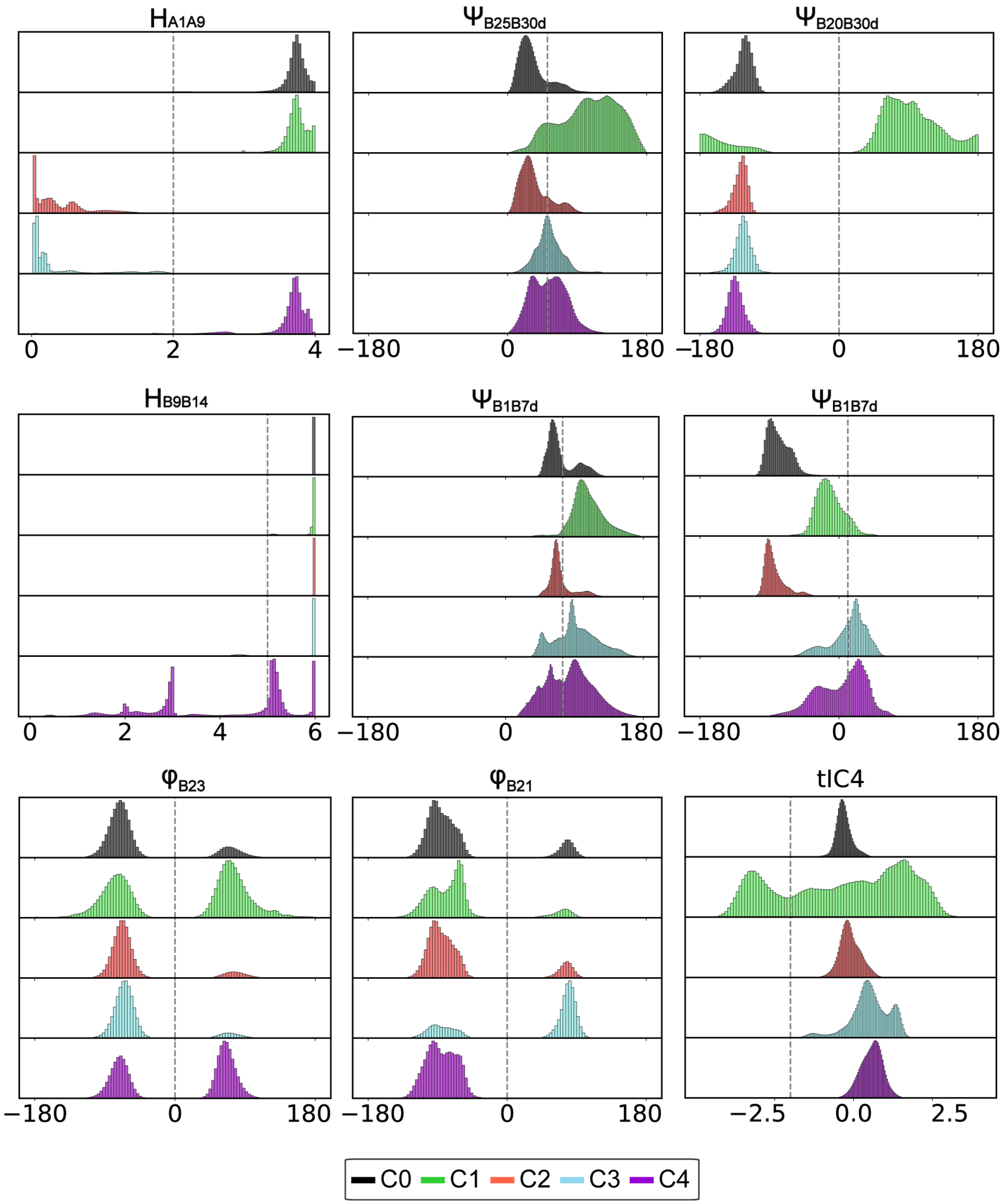

Figure S4: Probability density distribution of relevant CVs used to characterize structural heterogeneity in clusters. Vertical dashed line indicates the criterion used to categorize the presence/absence of disorder.

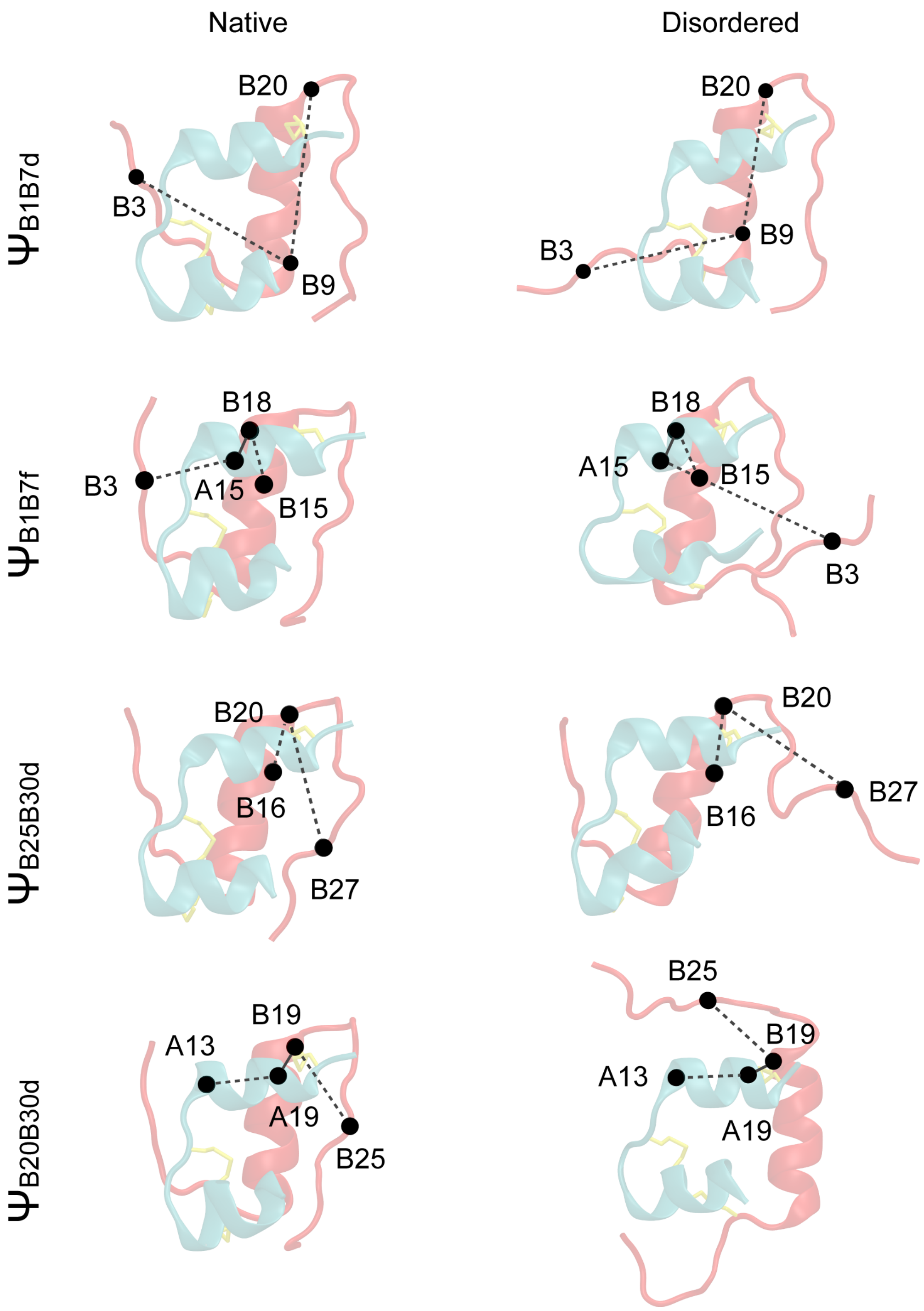

Figure S5: Points used to define Ψ_{B1B7d}, Ψ_{B1B7f}, $\Psi_{B25B30d}$, and $\Psi_{B20B30d}$.

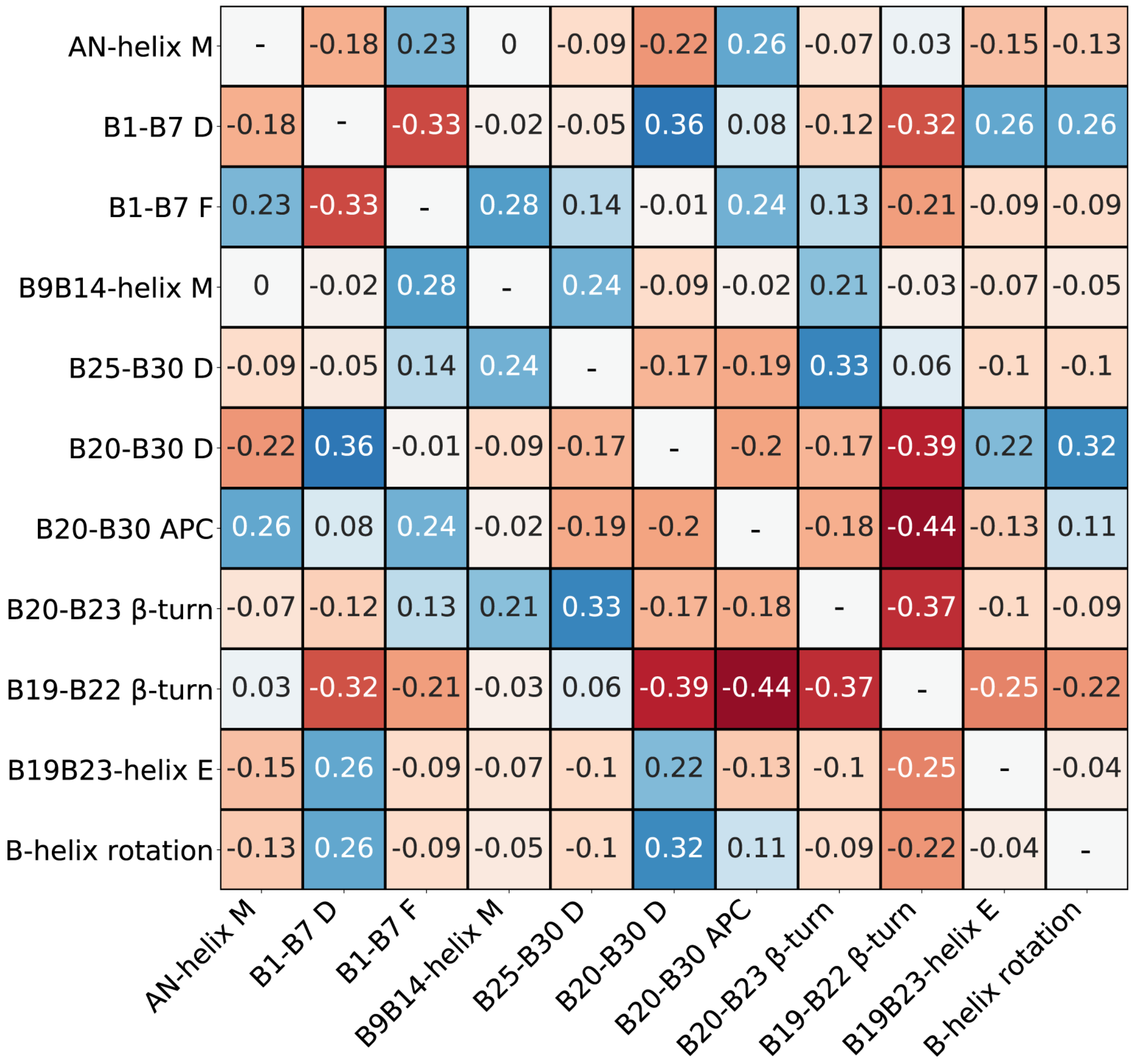

Figure S6: Correlation coefficient between elements of disorder in Table 1.

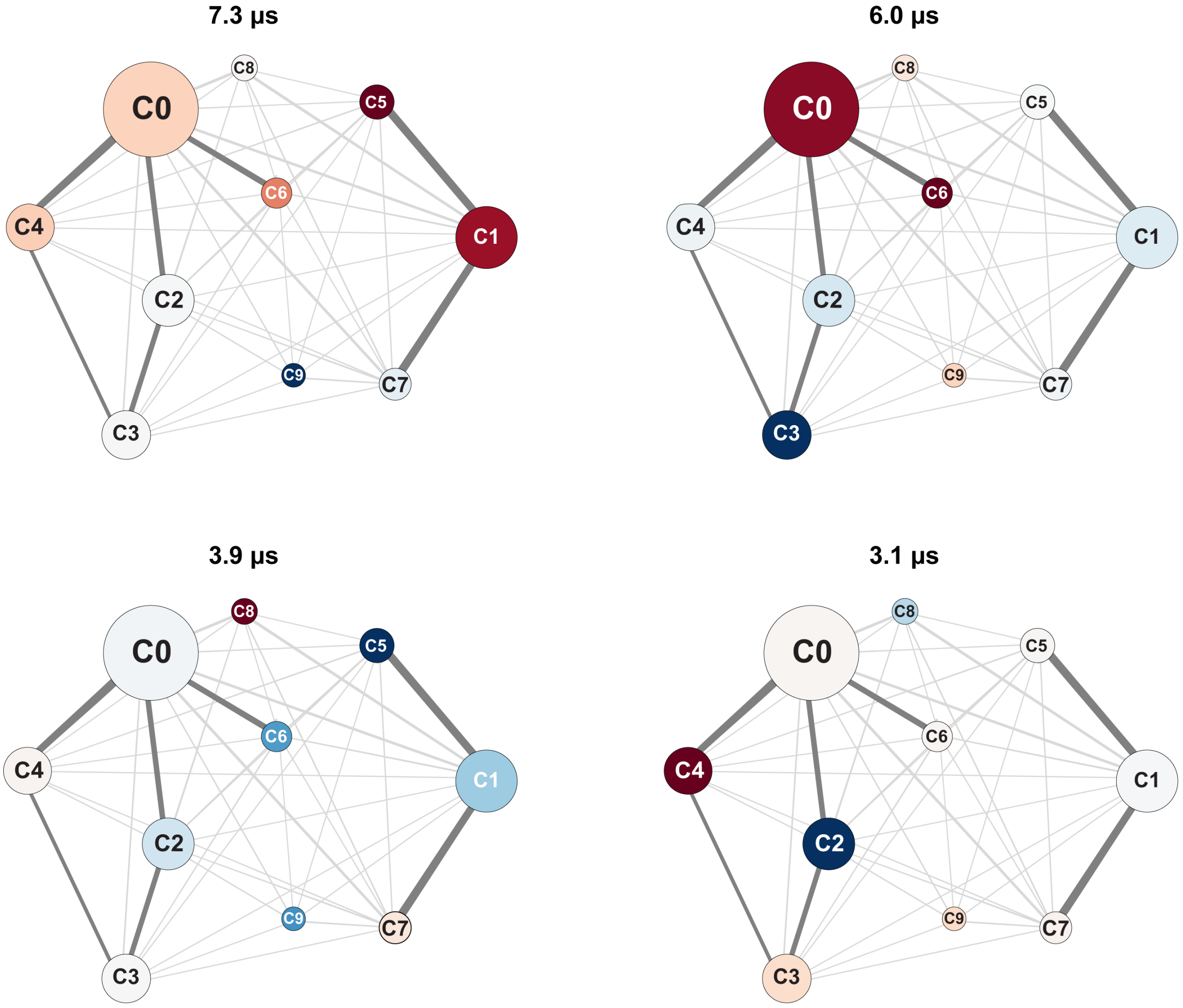

Figure S7: Network plots illustrating the fluxes between clusters 0 to 9, with the nodes color-coded according to the cluster mean value of second to fifth non-trivial eigenvectors of the MSM transition matrix. Eigenvectors were normalized to have a norm of 1. Zero is set to white. Corresponding implied time-scales are shown above.

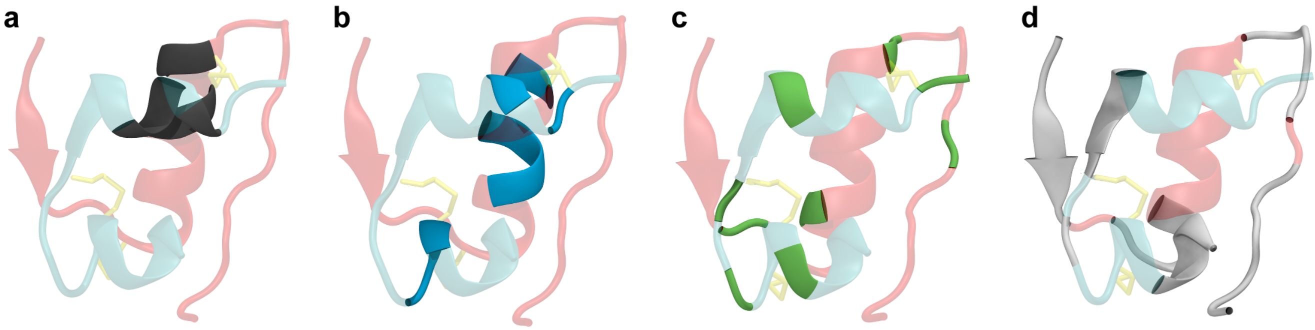

Figure S8: Structural map of protection factors reported in Fig 6. **(a)** Sites with $PF_i \geq 200$: A16, A17, A19, B15, B16, B18, and B19. **(b)** Sites with $200 > PF_i \geq 100$: A6-A8, A18, A20, B12-B14, B17. **(c)** Sites with $100 > PF_i \geq 50$: A5, A9, A11, A15, A21, B6, B11, B20, B25. **(d)** H_N sites with $50 > PF_i$: A1-A4, A10, A12-A14, B1-B5, B7-B10, B21-B24, B26-B30.

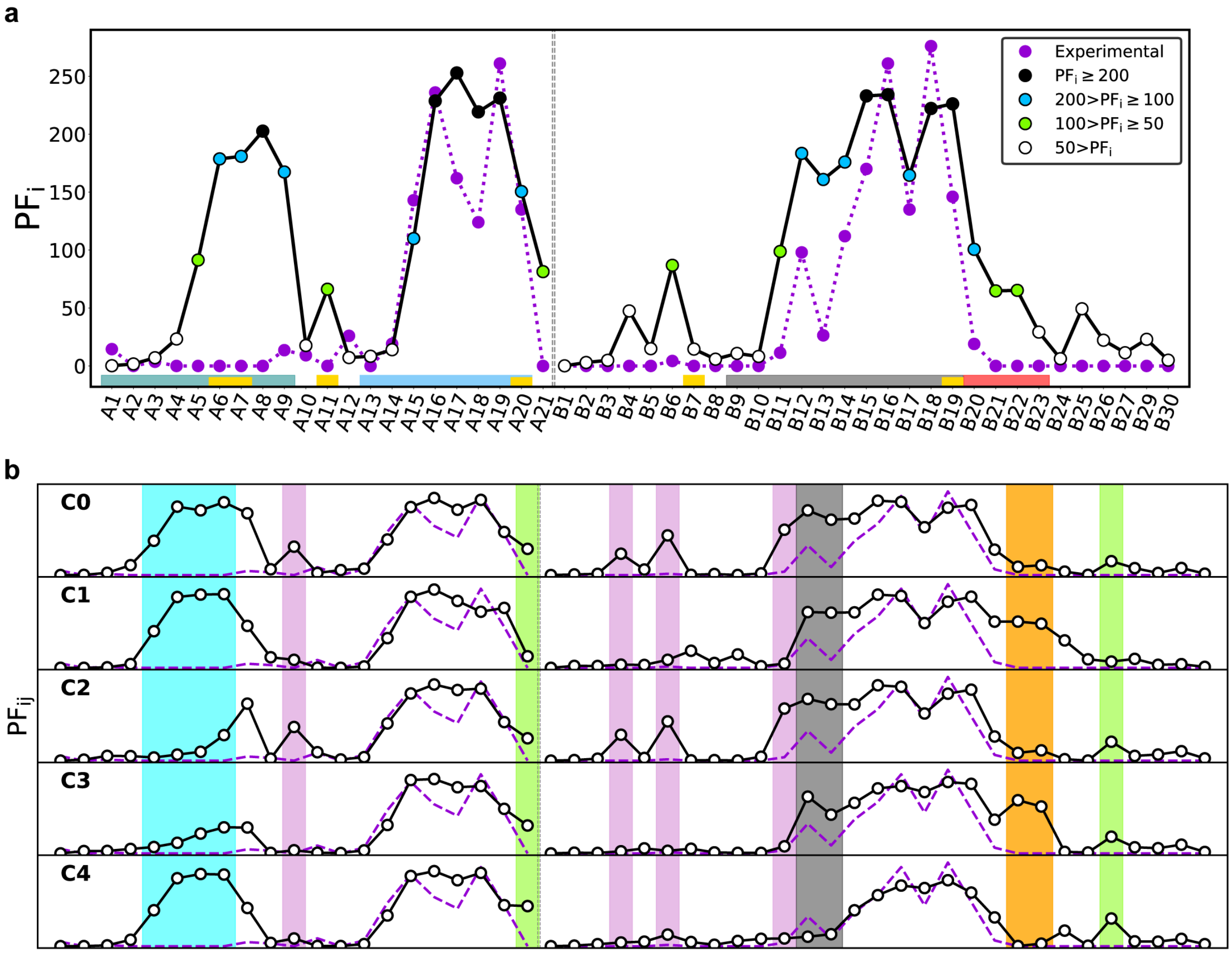

Figure S9: Protection factor prediction for the Ratio model, with hydrogen-bond angle and distance cutoffs of 70° and 0.5 nm, respectively. **(a)** Ensemble protection factor for each H_N site, taken as the weighted average for clusters 0 to 4. Different colors are used depending on the predicted PF (see legend). Experimental protection factors come from Hua *et al.*[5] Secondary structure motifs of insulin are indicated in the lower part as colored bars, following the color code of Fig 1. (**B**) Mean simulated protection factor (white circles) for clusters 0 to 4. Experimental values are plotted as a purple dashed line. Sites of interest are highlighted following the code in Figure 6.

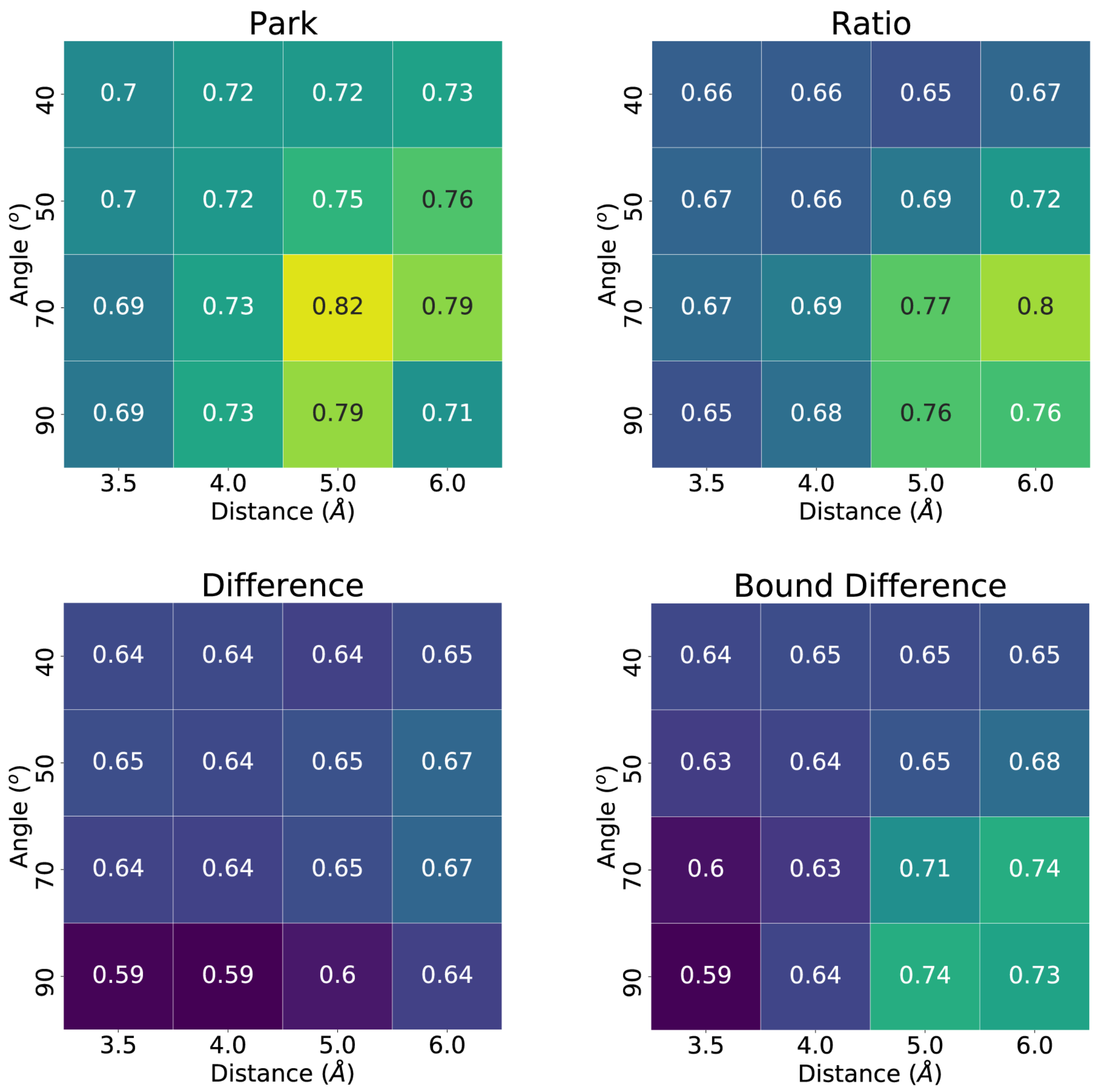

Figure S10: Correlation coefficients between experimental PFs and mean estimates using the four different models described in the Method section.

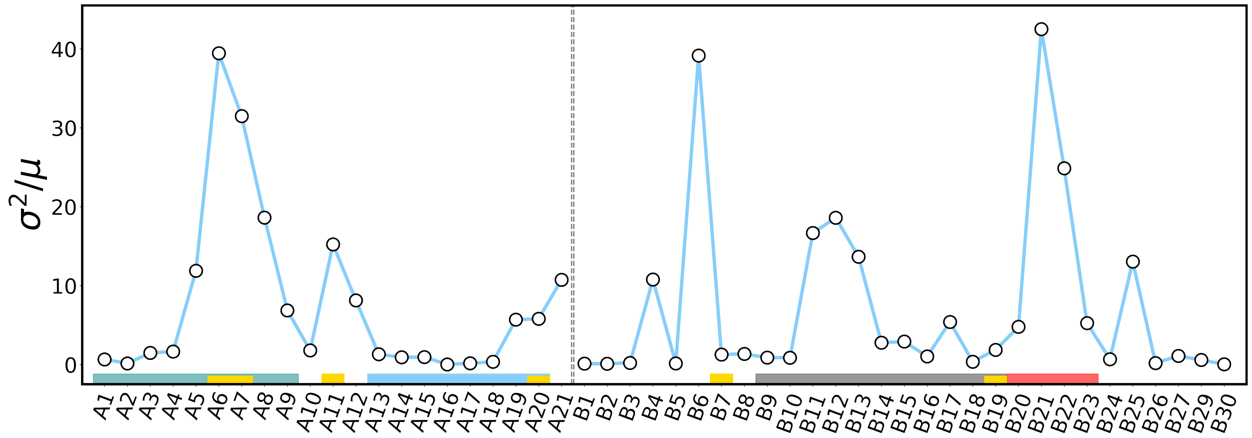

Figure S11: Variances relative to means (σ^2/μ) of the protection factors estimated with the Park model, with angle and distance cutoffs of 70° and 0.5 nm, respectively. Variances (σ^2) are computed across the 5 major clusters. Secondary structure motifs of insulin are indicated in the lower part as colored bars, following the color code of Fig. 1.

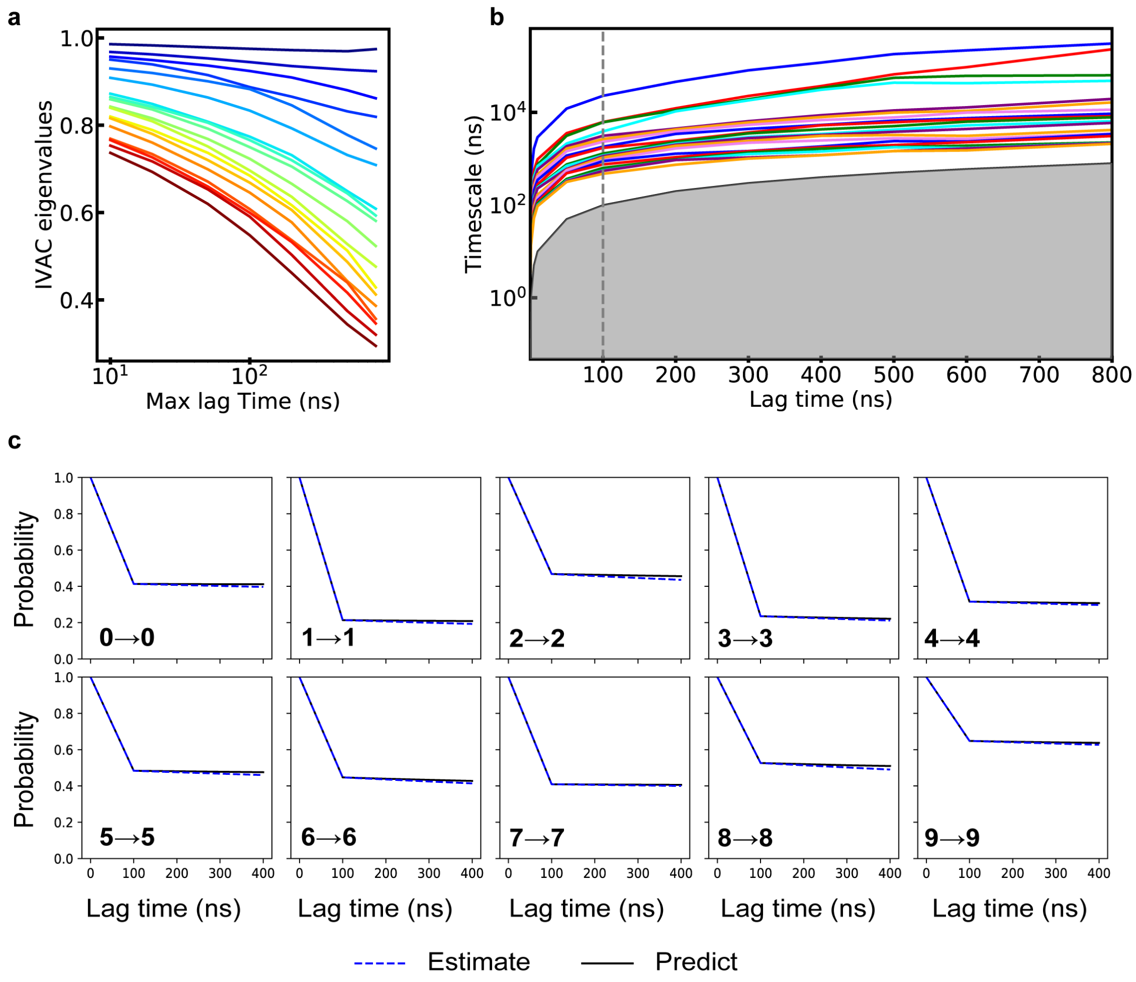

Figure S12: Validation of parameter choice for IVAC and MSM construction. **(a)** Eigenvalues associated with tICs as functions of lag time. **(b)** Implied time scales of the MSM as a function of lag time. Only the slowest 20 time scales are shown. Shadowed region corresponds to the area under the black curve defined by increasing lag times. Gray dashed line corresponds to the lag time chosen for the construction of the MSM (100 ns), which was found to be the shortest lag time satisfying the Chapman-Kolmogorov test (**c**). **(c)** Chapman-Kolmogorov test, as defined in PyEMMA 2.5.7.[6] For an MSM built at lag time τ, the test compares transitions probabilities between metastable states at time 5τ (black line), with an independently estimated MSM with lag time 5τ (blue dashed line). We only show self-transition probabilities for clusters 0 to 9.
